# Photo-Responsive In Situ Forming Hydrogels for Drug Delivery: A Critical Review of Polymer Matrices, Photoinitiators, and the Gap Towards Clinical Translation

**DOI:** 10.3390/polym18161951

**Published:** 2026-08-09

**Authors:** Elena O. Bakhrushina, Gleb A. Gribanov, Susanna S. Sologova, Hadi Darawsheh, Elkhan G. Osmanov, Elena A. Smolyarchuk, Yuriy L. Vasil’ev, Ivan I. Krasnyuk

**Affiliations:** 1 Department of Pharmaceutical Technology, A.P. Nelyubin Institute of Pharmacy, First Moscow State Medical University (Sechenov University), 119435 Moscow, Russia; 2 Department of Pharmacology, A.P. Nelyubin Institute of Pharmacy, First Moscow State Medical University (Sechenov University), 119435 Moscow, Russia; 3 Department of Topographic Anatomy and Operative Surgery, N.V. Sklifosovskiy Institute of Clinical Medicine, First Moscow State Medical University (Sechenov University), 119435 Moscow, Russia; 4 Department of Faculty Surgery No. 2 Named After G.I. Lukomsky, First Moscow State Medical University (Sechenov University), 119435 Moscow, Russia; 5 Lomonosov Institute of Fine Chemical Technologies, MIREA-Russian Technological University, 125993 Moscow, Russia

**Keywords:** photo-responsive hydrogels, in situ forming systems, drug delivery, photopolymerization, photoinitiators, GelMA, near-infrared light, Quality by Design, clinical translation

## Abstract

Local drug delivery increasingly relies on injectable hydrogels that form directly at the x‘administration site, among which light-cured systems are of particular interest: irradiation converts a liquid precursor into a depot and can trigger drug release with high spatiotemporal resolution. The aim of this critical narrative review is to systematize the key design elements of such systems and to assess their path toward the clinic. We analyze the mechanisms of photoactivation, the role of wavelength, the natural, synthetic, and hybrid polymer matrices together with their tuning parameters, and the photoinitiators. The clinical and preclinical experience in dentistry, ophthalmology, regenerative medicine, and oncology is then considered separately. Finally, we address standardization through the Quality by Design concept. We show that, despite an extensive preclinical base, no photocrosslinkable injectable depot for drug delivery has yet been approved, whereas photopolymerization itself and the photoinitiators employed are already accepted in the clinic in adjacent fields. We conclude that clinical translation is defined by three tractable tasks: qualifying photoinitiators for the injectable route of administration, overcoming the limited depth of light activation, and standardizing characterization on the basis of Quality by Design.

## 1. Introduction

Despite their widespread clinical use, conventional dosage forms retain a number of fundamental drawbacks, including systemic toxicity, non-specific distribution, the development of multidrug resistance, and rapid metabolic clearance. These problems are especially acute in oncology, where the narrow therapeutic window of cytostatic agents is limited by their non-selective cytotoxicity toward healthy proliferating cells [1,2]. It is precisely the inability of classical systems to distinguish healthy from pathological tissue that leads to suboptimal drug accumulation at the target and to dose-limiting toxicity. This fundamental limitation has, in turn, stimulated the development of stimulus-responsive platforms capable of providing spatiotemporal control over release [3].

The concept of in situ formation offers an elegant solution: the system is administered as a low-viscosity liquid, and the sol–gel transition occurs directly at the injection site, driven either by physiological conditions (temperature, pH, ionic composition of the biological fluid, enzymes) or by an external signal. Advances in polymer chemistry and materials science have made it possible to create formulations that can be injected through a standard fine needle, opening the way to minimally invasive delivery [4]. Gelation is typically achieved either through physical interactions (hydrophobic assembly, complexation) or through chemical crosslinking induced by light, enzymes, or chemical reagents. Notably, external stimuli provide more precise temporal control and help offset the inter-patient variability that is inevitable when endogenous triggers are used [5].

A key merit of such systems is the combination of minimal invasiveness with spatial adaptability. Unlike pre-formed implants, a liquid formulation fills irregularly shaped cavities, accurately conforming to their contours without mechanical damage to the surrounding tissue and without the contact failure characteristic of solid implants [6,7]. From a technological standpoint, these systems offer straightforward drug encapsulation, high loading capacity, and the potential to create depots with sustained release [8].

Among external stimuli, light stands out for its exceptional tunability: it is non-invasive, can be focused with high spatial resolution, and its parameters—wavelength, intensity, and exposure time—can be specified with high precision [9]. For in situ systems, this translates into direct operator control over the moment of gelation and the subsequent release kinetics, a level of control unattainable with endogenous triggers. The basic feasibility of this approach has already been demonstrated in experiments in mice [10]. Serious limitations nevertheless remain. Ultraviolet (UV) radiation, traditionally used for photoinitiation, raises concerns because of its phototoxicity and shallow penetration depth (<1 mm). As an alternative, visible-light systems (for example, those based on eosin Y) have been proposed; these are safer, although penetration depth remains a bottleneck. In this context, the near-infrared (NIR) range (650–950 nm) is attracting growing attention, since it is markedly less toxic and penetrates to a depth of 1–3.5 mm [11,12].

The aim of this review is to systematize approaches to the design of photo-responsive in situ delivery systems, with a primary focus on the materials-science foundations of their design. In contrast to earlier broad reviews, we concentrate specifically on in situ gel-forming platforms. The article is organized as follows. Section 3 addresses the mechanisms of photoactivation. Section 4 provides a critical analysis of polymer matrices. Section 5 evaluates photoinitiators and their influence on gelation kinetics, mechanical properties, and cytotoxicity. Section 6 summarizes data on the use of such systems for drug delivery at the preclinical and clinical levels. In the concluding Section 7, the key translational barriers are identified—qualification of initiators, depth of light penetration, and the lack of standardization—and the Quality by Design (QbD) concept is proposed as a tool for addressing them systematically.

## 2. Materials and Methods

This work is a critical narrative review of photo-responsive in situ forming hydrogels for drug delivery. The literature search was conducted in the PubMed, Scopus, Web of Science, and Google Scholar databases. The clinical and regulatory status of the technologies was assessed using data from ClinicalTrials.gov, official FDA resources, and ISO standards and ICH guidelines.

Priority was given to peer-reviewed publications from 2020 to 2026; seminal earlier works were included selectively. Combinations of English-language keywords were used (including “photo-responsive hydrogel”, “in situ forming hydrogel”, “photopolymerization”, “photoinitiator”, “GelMA”, “drug delivery”, and “Quality by Design”) with the Boolean operators AND/OR. The review included original research articles and reviews on photocrosslinkable in situ hydrogels and their components, as well as sources addressing their clinical and regulatory status.

The following information was extracted from the sources: the polymer matrix and crosslinking chemistry, the photoinitiator and wavelength, the study model, and the key results; the data were organized into thematic sections and compiled into comparative tables that distinguish approved, clinical, and preclinical systems. This work is a review and contains no new experimental data: all analyzed information was obtained from published sources listed in the references with their DOIs and PMID/PMCID identifiers.

## 3. Mechanisms of Photoactivation

### 3.1. General Classification of Photoinduced Effects

**Figure 1:**
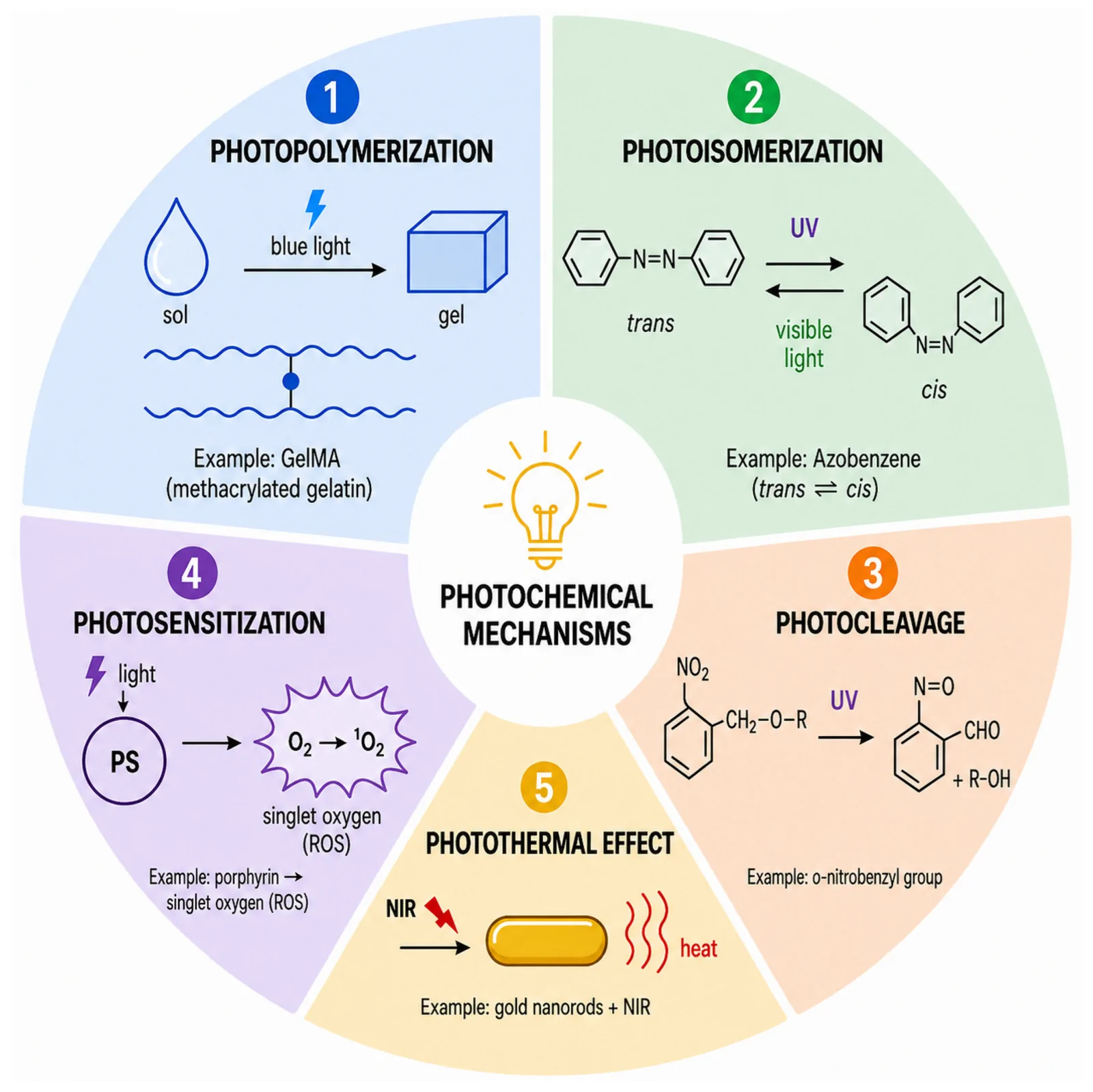
Principal photochemical mechanisms of light-responsive in situ delivery systems.

Light-responsive delivery systems operate through several distinct photochemical mechanisms [13]. In in situ systems, the roles of these mechanisms differ: photopolymerization (photocrosslinking) is responsible for the formation of the gel itself from the injected precursor solution, whereas release from an already-formed light-responsive hydrogel proceeds predominantly through photoisomerization, photochemical reactions, and the photothermal effect [9,10]. In a systematic review, Zhang et al. (2025) identified the main types of mechanisms in photoactive delivery systems—photopolymerization, photoisomerization, photocleavage, photosensitization, and photothermal phenomena (Figure 1) [13].

Light sensitivity in delivery systems is usually conferred by introducing photoactive compounds—either into the polymer matrix (into the backbone or as pendant substituents) [14]. Based on the nature of the photochemical response, two main classes are distinguished. Photochromic groups (azobenzenes, spiropyrans, diarylethenes) isomerize reversibly upon irradiation, accompanied by changes in polarity and hydrophobicity [15]. Photolabile groups (derivatives of o-nitrobenzyl and coumarin), by contrast, undergo irreversible bond cleavage [15].

Which of these mechanisms proves decisive depends on what is required in practice: to form a gel or to trigger release from an existing gel. For in situ gelation, the key process is photopolymerization (or photocrosslinking)—it converts the injected liquid precursor solution into a solid hydrogel. Once the gel has formed, drug release can be further modulated using photoisomerization, photochemical cleavage, photosensitization, or photothermal effects [9,10,13]. Because photopolymerization determines the success of in situ formation, the central engineering question is how the reactive species (radicals or cations) are generated. According to the origin of these species, photocrosslinkable in situ systems fall into two broad categories: self-activating systems (in which photoactive groups are covalently attached to the polymer) and systems that depend on a separately added photoinitiator. Each is discussed in more detail below.

### 3.2. Self-Activating Systems (Photoactive Polymers)

In self-activating systems, light sensitivity is built directly into the polymer chain or its pendant substituents. The classic approach is photodimerization: coumarin, cinnamate, or anthracene groups serve as reversible crosslinking points. Upon irradiation they dimerize, leading to gelation, whereas the reverse photocleavage reaction can be used to reduce crosslinking density [16].

The main advantage of this approach is compositional simplicity. Gelation requires only a single polymer precursor, with no need for an additional photoinitiator, reactive monomers, crosslinking agents, or—importantly—potentially toxic additives [17]. These systems are not, however, a universal solution. Photodimerization typically requires UV radiation: coumarin dimerizes at around 365 nm, while the reverse cleavage of crosslinks requires short-wavelength light of about 254 nm, which is poorly suited to biological settings because of its phototoxicity and shallow penetration depth [16].

### 3.3. Photoinitiator-Dependent Systems

Most photocrosslinkable matrices used in practice—such as gelatin methacryloyl (GelMA), poly(ethylene glycol) diacrylate (PEGDA), and their analogs—do not crosslink under light on their own. They require a photoinitiator: a light-sensitive compound that, upon absorbing photons, generates reactive species—most often free radicals [18]. These radicals attack the reactive groups (acrylate, methacrylate) on the polymer chains, converting them into new radicals that then participate in chain growth and ultimately form a crosslinked polymer network [19].

In other words, the photoinitiator acts as a molecular transducer that converts a light signal into a chemical crosslinking reaction. Without it, light alone—at least at biocompatible doses—is usually unable to trigger gelation. The choice of photoinitiator determines the activation wavelength, the required irradiation dose, and, to a large extent, the biocompatibility of the resulting gel. Initiator selection is therefore not a secondary detail but one of the central elements of system design. A detailed classification and comparative assessment of the most common photoinitiators are provided in Section 5.

### 3.4. Wavelength as a Key Design Parameter

Activation wavelength is a cross-cutting parameter that ties together the choice of photoinitiator, biosafety, and the achievable penetration depth. Short-wavelength UV radiation (250–350 nm) not only damages biological structures directly but also penetrates tissue to a depth of less than 1 mm [20]. Visible light is safer, yet its depth is also limited. By contrast, the near-infrared range (650–900 nm) penetrates considerably deeper—up to several centimeters—and causes minimal tissue damage [21]. These considerations have driven a logical shift from UV to visible light and, increasingly, to NIR.

**Figure 2:**
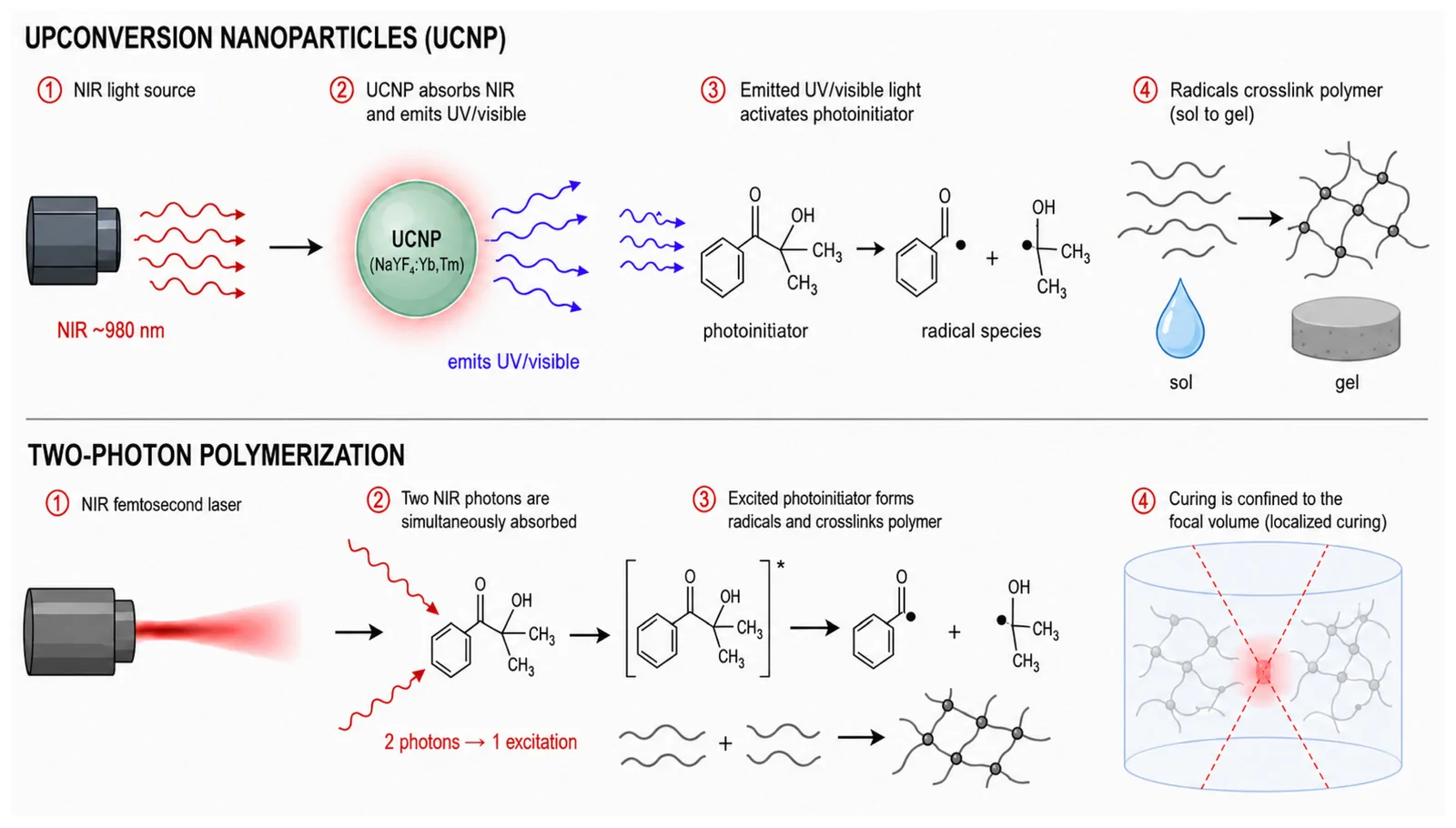
Near-infrared activation of photopolymerization using upconverting nanoparticles (UCNPs) and two-photon polymerization. The asterisk (*) denotes the electronically excited state of the photoinitiator.

The move to visible light is achieved mainly by selecting an appropriate photoinitiator (Section 5). Activation in the NIR range, however, is technically more challenging, because low-energy photons cannot directly initiate photochemical reactions. Two main strategies have emerged here: two-photon polymerization and the use of upconverting nanoparticles (UCNPs). UCNPs (a typical composition being NaYF_4_:Yb^3+^,Tm^3+^) absorb NIR radiation and re-emit it at shorter (UV or visible) wavelengths, which then excite the photoinitiator and initiate polymerization (Figure 2) [20].

The basic applicability of this approach for in situ systems has been confirmed in two distinct experimental scenarios. In one study, UCNPs incorporated into the precursor solution converted NIR that had passed through tissue into blue light, triggering gelation directly within the tissue [22]. In another example, upconverted UV radiation cleaved photocrosslinkable bonds in a hydrogel placed beneath a two-centimeter layer of tissue, allowing drug release to be switched on and off simply by controlling the NIR source [23].

The discussion in Section 3.2, Section 3.3 and Section 3.4 thus leads to a practical conclusion: a photo-responsive in situ system can be designed rationally by combining a suitable polymer matrix with an appropriate mode of photoactivation. The developer either incorporates photoactive groups into the polymer itself (Section 3.2) or selects an external photoinitiator (Section 3.3) with the required wavelength (Section 3.4). This modularity means that the design task reduces to two decisions—the choice of polymer and the choice of photoinitiator—which are the subject of Section 4 and Section 5 respectively.

## 4. Selection of the Polymer Matrix

### 4.1. Design Criteria for Photocrosslinkable Polymers

A polymer intended for photocrosslinkable in situ systems must meet several requirements, some of which are common to any injectable in situ formulation and some of which are specific to photoactivatable platforms.

First, the polymer must be biocompatible and biodegradable; here, natural biopolymers hold a clear advantage [8]. Second, the initial formulation must remain liquid (in the sol state) at the time of administration and crosslink under light under mild physiological conditions; indeed, mild crosslinking conditions and the possibility of minimally invasive delivery are key merits of photocurable matrices [24]. The third requirement is the presence of, or the ability to introduce, reactive groups. Natural polymers are convenient in that they already contain chemical groups amenable to functionalization: in hyaluronic acid these are the primary hydroxyl and carboxyl groups [25], and in chitosan the amino groups, which allow methacrylate groups to be introduced and thereby confer photocrosslinkability [26].

In addition, controllable degradation is required: the rate of gel breakdown must match the intended application (for drug delivery, the release profile and clearance), and this rate must be tunable by chemical modification [27,28]. Closely related is the fifth requirement—the ability to tune mechanical properties and gelation kinetics. The principal control parameter here is the degree of substitution (DS) with reactive groups, together with macromer concentration and molecular weight [24,29]. Finally, photosystems carry a specific condition: the crosslinking process itself (light dose, type and concentration of photoinitiator, and the reactive oxygen species generated) must not damage cells or tissue. This issue is addressed in detail in Section 5.

### 4.2. Natural Polymers

Before turning to specific examples, one point deserves emphasis: most natural polymers are not intrinsically photoactive. Photocrosslinkability is conferred on them through chemical functionalization, most often methacrylation. The leading natural platforms are therefore methacryloyl derivatives.

#### 4.2.1. Gelatin Methacryloyl (GelMA)

This is the most extensively studied of all photocurable biopolymers. Gelatin is denatured collagen and, importantly, retains bioactive sequences of the native protein. Methacrylation affects neither the RGD motifs (responsible for cell adhesion) nor the sites recognized by matrix metalloproteinases (MMPs), so GelMA reproduces many properties of the extracellular matrix (ECM) [25]. The gel forms under UV or visible light in the presence of a photoinitiator, and its properties are tuned through the degree of methacryloyl substitution. It is precisely this combination of biocompatibility, bioactivity, and processability that has made GelMA a widely used material in the field of photocurable hydrogels [12].

#### 4.2.2. Gelatin Norbornene (GelNB)–A Thiol–ene Alternative

Alongside methacrylation, gelatin can be functionalized with norbornene groups to yield GelNB. Norbornene undergoes a thiol–ene photoclick reaction with thiol-bearing crosslinkers (for example, dithiothreitol or PEG-tetrathiol) under visible or long-wavelength UV light [30]. The fundamental difference from GelMA lies in the type of polymerization. Methacrylate polymerization proceeds by a chain-growth mechanism, whereas the thiol–norbornene reaction is a step-growth polymerization. In practice, this yields a more homogeneous network with less shrinkage, insensitivity to oxygen inhibition, and milder crosslinking conditions. Accordingly, GelNB is increasingly in demand where high precision and maximal cytocompatibility are critical.

#### 4.2.3. Hyaluronic Acid and Its Methacrylated Derivatives (MeHA or HAMA)

Hyaluronic acid (HA) is a natural glycosaminoglycan that becomes photocrosslinkable after methacrylation [25]. However, the principal value of HA lies not in its mechanical properties but in its biological function: it serves as an endogenous ligand for the CD44 receptor, which is overexpressed in many solid tumors. MeHA-based systems thus possess built-in tumor targeting. An important practical note is that the degree of methacrylate substitution creates a trade-off. A high degree of modification (on the order of 40%) increases crosslinking density but, as reported in the literature, reduces affinity for CD44 [31]. In designing such systems, one must therefore balance the mechanical stability of the gel against retention of its targeting properties.

Beyond methacrylation, HA can be crosslinked through other chemistries that broaden its property range [C31]. The Diels–Alder [4+2] cycloaddition between furan-modified HA and a maleimide crosslinker forms reversible covalent bonds between furan and maleimide groups under mild aqueous conditions, without a photoinitiator, yielding injectable, self-healing networks [32]. Notably, this chemistry pairs well with light—furan-modified HA has been gelled within ~30 s by LAP-mediated photocrosslinking and then reinforced by a thermal Diels–Alder reaction, raising the modulus from ~5–11 to ~21 kPa while preserving encapsulated-cell viability [33]. Alternatively, dynamic-covalent Schiff-base (imine), hydrazone, and oxime bonds formed on aldehyde-modified HA yield injectable, self-healing networks under mild physiological conditions [34].

#### 4.2.4. Chitosan

This polymer, obtained by deacetylation of chitin, is known for its mucoadhesiveness, low cytotoxicity, biodegradability, and antibacterial and hemostatic properties [35]. Its use in in situ systems is nevertheless limited by poor solubility at physiological pH. A solution was found in the use of a derivative–glycol chitosan, which is soluble at neutral pH and becomes photopolymerizable after methacrylation with glycidyl methacrylate [36]. Methacrylated chitosan exhibits enhanced mucoadhesion, making it attractive for transmucosal delivery [37]. Tellingly, a photocrosslinkable in situ gel based on glycol chitosan simultaneously provides tissue adhesion, hemostatic and antibacterial activity, and controlled drug release [35]. Systems with a dual crosslinking mechanism (thermal and photochemical) have also been developed, in which additional physical crosslinking increases gel stability. Allylation of chitosan enables photocrosslinkable compositions with synthetic polymers that, after laser stereolithography, retain biocompatibility and support the adhesion and proliferation of mesenchymal stromal cells [27].

#### 4.2.5. Collagen

As the most abundant protein of the extracellular matrix, collagen offers high biocompatibility, biodegradability, and cell-adhesion domains [28]. Native collagen, however, has two limitations that are critical for in situ systems: very low stiffness and rapid enzymatic degradation [38]. Photocrosslinking overcomes both problems. Notably, riboflavin (vitamin B_2_)—a biocompatible compound with minimal cytotoxicity—is often used as the photoinitiator for collagen [38]. The advantage of the riboflavin-mediated approach is that it allows the gel properties to be controlled while preserving the native biochemical environment of collagen without intensive chemical modification [28]. It should be noted that gelatin (the basis of GelMA) is a denatured form of collagen, so collagen and GelMA constitute a single family of collagen-based materials.

Crosslinking of collagen is likewise not limited to riboflavin-mediated systems. Collagen can be made directly photocrosslinkable by methacrylating its backbone: methacryloyl functionalization has a negligible effect on triple-helix stability, and the resulting shear-thinning, injectable precursor is photocrosslinked under UV light while keeping encapsulated cells viable [39]. As a visible-light alternative to the riboflavin/UV-A system, rose bengal activated by green light (RGX) photochemically crosslinks collagen, improving its otherwise poor mechanical stability and enabling controlled release of incorporated substances [40].

#### 4.2.6. Gellan Gum and Its Methacrylated Derivatives (GGMA/MeGG)

Gellan gum (GG) is a naturally occurring polysaccharide that crosslinks physically in the presence of divalent cations but physically crosslinked gellan gum hydrogels lose stability under physiological conditions [41]. In its native form it is both thermo- and ionic-responsive and is structurally similar to glycosaminoglycans, which makes it attractive for cartilage-oriented scaffolds [42,43]. To render it photocurable, methacrylate groups are introduced onto its carboxyl and hydroxyl groups using methacrylic anhydride or glycidyl methacrylate, giving methacrylated gellan gum (GGMA/MeGG) that can be crosslinked by chain-growth photopolymerization, by thiol–ene photoclick chemistry, or by a combination of the two [41]. Its compressive modulus (~6–17 kPa), swelling, and enzymatic (lysozyme) degradation are tuned by the degree of methacrylation, the thiol–ene ratio, and the amount of added calcium, which both stiffens the network and suppresses swelling [41]. These features suit GGMA to in situ use: the low-viscosity precursor withstands injection shear and crosslinks rapidly on contact with physiological fluid while keeping encapsulated cells viable [R3]. Its thermal, ionic (Ca^2+^/Mg^2+^), and photochemical crosslinking can be combined orthogonally to tune network strength and gelation timing largely independently [44]. Directly relevant to the present review, injectable GGMA has been crosslinked in situ under visible light using the cytocompatible ruthenium/sodium persulfate system, supporting embedded human chondrocytes with high viability [R1]. Like other physically gelling polysaccharides, GG therefore relies on this covalent methacrylate reinforcement to obtain stable, load-bearing networks [41,44].

#### 4.2.7. Other Natural Polymers (Alginate, Fibrin)

Under ordinary conditions, alginate crosslinks with calcium ions, but after the introduction of methacrylate groups it acquires the ability to respond to light. This makes it possible to form photocrosslinked gels with tunable degradation rate and mechanical properties by varying the degree of methacrylation [24]. Oxidation of alginate combined with methacrylation (OMA) provides an even broader range of controllable degradation. Fibrin can be used via methacrylated fibrinogen (FibMA) [18]. Although these polymers are used less frequently, they expand the available toolkit of materials for photocurable systems.

### 4.3. Synthetic Polymers

Whereas natural polymers provide bioactivity and cues for cells, synthetic polymers offer reproducibility, a chemically defined composition, and precisely tunable properties. The flip side of this is, as a rule, bioinertness: synthetic polymers lack intrinsic cell-adhesion cues. Natural and synthetic matrices therefore do not so much compete as complement one another.

#### 4.3.1. PEGDA (Poly(ethylene glycol) diacrylate)

PEGDA is a widely recognized benchmark of bioinertness. It consists of a poly(ethylene glycol) (PEG) chain bearing acrylate groups at both ends. These double bonds allow PEGDA to participate in free-radical photopolymerization in the presence of a photoinitiator. PEG itself is a hydrophilic, water-soluble, biocompatible polymer with low protein adsorption, which accounts for its “invisibility” to the immune system [45]. PEGDA is non-toxic, elicits a minimal immune response, and biodegrades through hydrolysis of the ester acrylate bonds [46].

The key control parameter is the molecular weight of PEGDA. The lower the molecular weight, the higher the crosslinking density and the lower the swelling. High-molecular-weight systems yield a looser network and, correspondingly, accelerated drug release [46]. This dependence has been confirmed quantitatively: the release of a model protein from PEGDA gels correlates directly with macromer molecular weight and concentration [47]. The main technological limitation of PEGDA is oxygen inhibition of acrylate polymerization, as well as the need for additional functionalization (for example, with RGD peptides) to provide cell adhesion.

#### 4.3.2. Poloxamers (Pluronics) with Photoactive Modifications

Poloxamers are PEO–PPO–PEO triblock copolymers known for their thermoresponsiveness. Poloxamer 407 is included in the pharmacopeias and in the FDA Inactive Ingredient Database, attesting to its recognized safety. However, physical poloxamer gels have low mechanical strength and are rapidly washed out. Introducing acrylate groups substantially improves the situation: diacrylated Pluronic F127 (DA-PF127) combines the intrinsic thermoresponsiveness with covalent photocrosslinking, markedly increasing gel stability compared with the unmodified analog [48]. This combination of two mechanisms (thermal and photochemical) is especially convenient for injectable systems: the formulation can be administered as a thermogel (which already provides initial fixation) and then subjected to additional photocrosslinking to achieve final stability.

#### 4.3.3. PLA and PLGA as Biodegradable Blocks

Polylactide (PLA) and its copolymer with glycolide (PLGA) are valued for their predictable hydrolytic degradation. In photo-based systems they are used not as standalone photo-matrices but as biodegradable segments within more complex macromers. The hydrolytic degradation rate of the whole system is governed by the composition and proportion of the PLA/PGA segments, in particular by the lactide-to-glycolide ratio. This makes them a convenient, tunable “degradable module” [49]. PLGA is also used in thermosensitive copolymers such as PLGA-PEG for in situ gelation [50]. That route, however, is no longer a photo-based system in the strict sense. Thus, PLA/PLGA serve rather as a “degradable module” that is incorporated into a photocrosslinkable construct.

#### 4.3.4. Poly(vinyl alcohol) (PVA), Poly(acrylic acid) (PAA), and Polyacrylamide (PAM)

These synthetic polymers also form part of the researcher’s toolkit. PVA is rendered photocrosslinkable by attaching reactive groups to its hydroxyl chains. Such PVA hydrogels form within minutes at physiological pH and temperature under long-wavelength UV in the presence of a non-toxic photoinitiator; being intrinsically non-adhesive to cells, they can be functionalized with the RGDS peptide [51]. PAA introduces pH sensitivity through its ionizable carboxyl groups and is readily (meth)acrylated, although on its own it has low mechanical strength [52]. PAM, formed by radical polymerization of acrylamide, is by definition compatible with photoinitiated crosslinking. In practice, these polymers are more often encountered as components of copolymer or interpenetrating networks (IPNs), where they impart either pH sensitivity or the required mechanical characteristics.

### 4.4. Hybrid and Emerging Systems

The rationale for building hybrid systems is to combine the advantages of natural and synthetic components. The natural polymer provides bioactivity (RGD motifs, mucoadhesion, cues for cells), while the synthetic polymer provides tunable mechanical properties and bioinertness. Blending GelMA with other polymers is one of the most attractive strategies. For example, combining PEGDA and GelMA as an interpenetrating network (IPN) allows porosity and mechanical properties to be tuned simply by changing the GelMA concentration [53]. The same approach works for the chitosan/PEG pair. IPNs often combine two types of crosslinking—for example, ionic and photochemical—which provides an additional degree of freedom in tuning rheological properties and porosity [53].

#### 4.4.1. Nanocomposite Matrices for NIR Activation

These are used for photo-controlled release in deep tissue layers. The idea is to introduce into the hydrogel nanoparticles that absorb NIR and convert it into heat. An injectable nanocomposite gel containing gold nanorods, for example, is simultaneously NIR- and pH-responsive, provides a photothermal response, and enables doxorubicin release over more than a month in a chemo-photothermal therapy regimen [54]. Besides gold, black phosphorus is used: photothermal therapy based on such nanoagents under NIR irradiation is notable for its simplicity, minimal invasiveness, and high spatiotemporal control [55]. This category also includes the UCNP-containing hybrids mentioned above (Section 3.4), which enable photochemical reactions to be activated by NIR light at depth. Importantly, such systems provide a dual therapeutic effect: not only controlled release but also photothermal therapy, which is especially valuable in oncology.

#### 4.4.2. 4D-Printable Photo-Responsive Hydrogels

The term “4D” in this context denotes a programmed change in shape over time in response to an external stimulus. Photocrosslinking here serves as a fabrication method via 3D printing (SLA, DLP, or two-photon polymerization), with the fourth dimension being the change in shape or volume after printing. Hydrogels capable of changing their properties in response to stimuli provide high specificity, controllability, and spatiotemporal resolution [56]. Applied to drug delivery, this opens the way to shape-adaptive depots and implants. In [57], composite hydrogels based on alginate and methylcellulose were developed that possess rheological properties suitable for extrusion 4D printing and the ability to retain a prescribed geometry after deposition. By varying the material-deposition trajectory and the orientation of the printed layers, the authors created structural anisotropy, which—upon subsequent ionic crosslinking of the alginate and swelling—produced non-uniform deformation. As a result, initially flat specimens were transformed in a programmable manner into helices, tubular structures, and other three-dimensional constructs.

Table 1 presents the comparative characteristics of the key polymer platforms used to create photocurable in situ hydrogels, highlighting their origin, crosslinking chemistry, biological feature, and tuning parameter. Table 2 compares the same platforms from a practical standpoint—mechanical properties, degradation, injectability, drug-loading capacity, photocuring conditions, and clinical maturity, together with their main advantages and limitations.

## 5. Selection and Characterization of Photoinitiators

### 5.1. Classification of Photoinitiators

Photoinitiators are classified primarily by their mechanism of radical generation. Two main types are distinguished—type I (cleavage-type) and type II (electron-transfer)—each with its own features relevant to biomedical applications [25]. In practice, two further important groups are added to these two basic classes: metal-containing (ruthenium) photocatalysts and nature-inspired initiators; mechanistically these resemble type II but stand out because of their particular properties. Across all groups, the cross-cutting practical parameter remains the activation wavelength (see Section 3.4).

#### 5.1.1. Type I Photoinitiators (Radical Cleavage, α-Scission)

**Figure 3:**
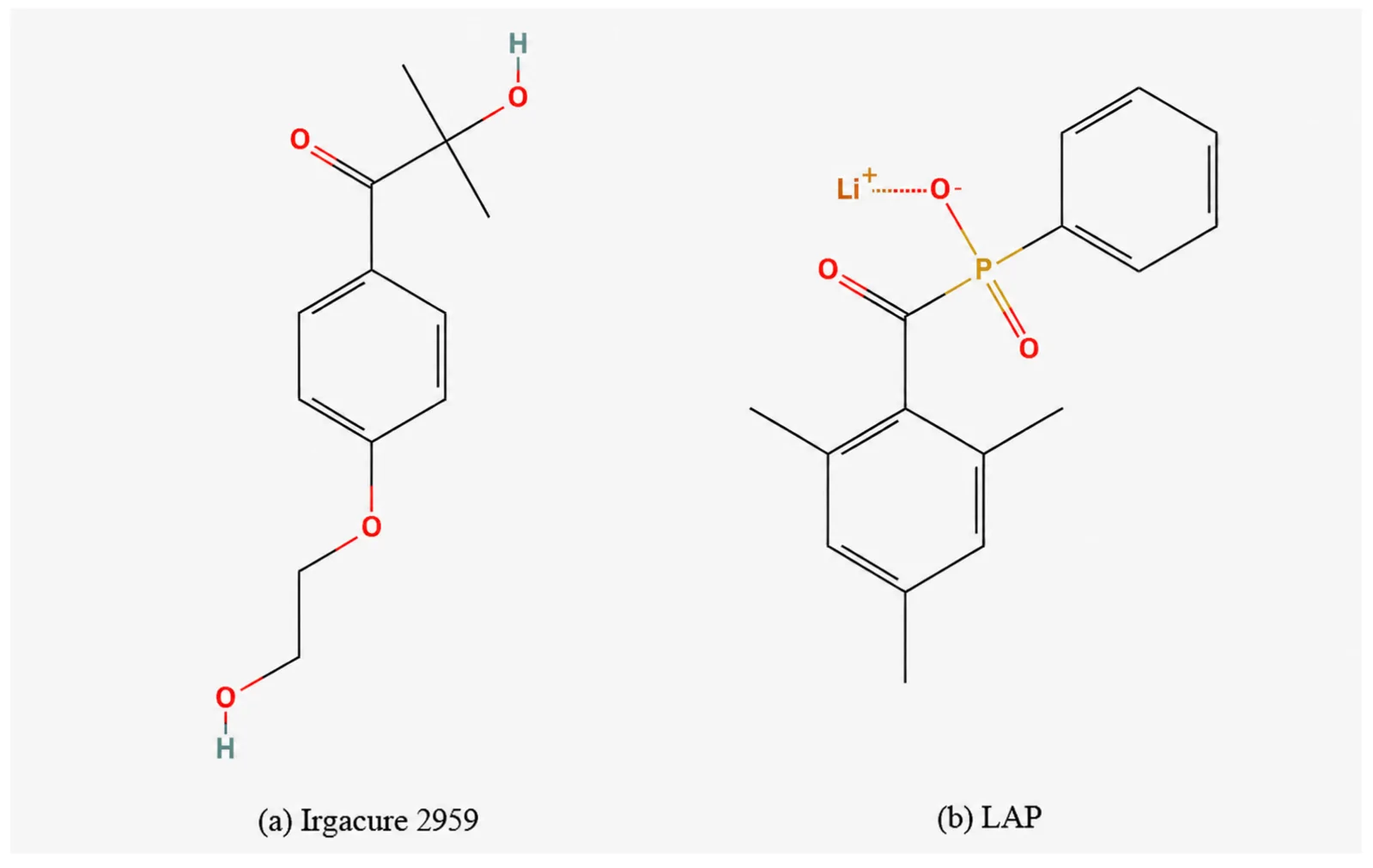
Representative Type I photoinitiators: (**a**) Irgacure 2959 and (**b**) LAP.

Typical representatives are Irgacure 2959 (I-2959) (Figure 3a) and lithium phenyl-2,4,6-trimethylbenzoylphosphinate (LAP) (Figure 3b). Initiators of this type generate radicals by direct photolytic cleavage of a labile bond under light, producing two reactive radical species at once without the need for a co-initiator [18]. Within the class, initiators differ substantially in activation wavelength: I-2959 is activated by UV light (365 nm), whereas LAP is activated by light of 405 nm [18]. This difference is fundamental: I-2959 remains one of the most common initiators, yet its absorption peak lies around 280 nm, and the associated need for prolonged UV-A irradiation can produce cytotoxic effects, prompting a search for safer photopolymerization strategies [58]. LAP is preferable in this respect: it is water-soluble, absorbs at a longer wavelength, and provides faster, milder crosslinking.

#### 5.1.2. Type II Photoinitiators (Bimolecular Mechanism, Photosensitizers)

**Figure 4:**
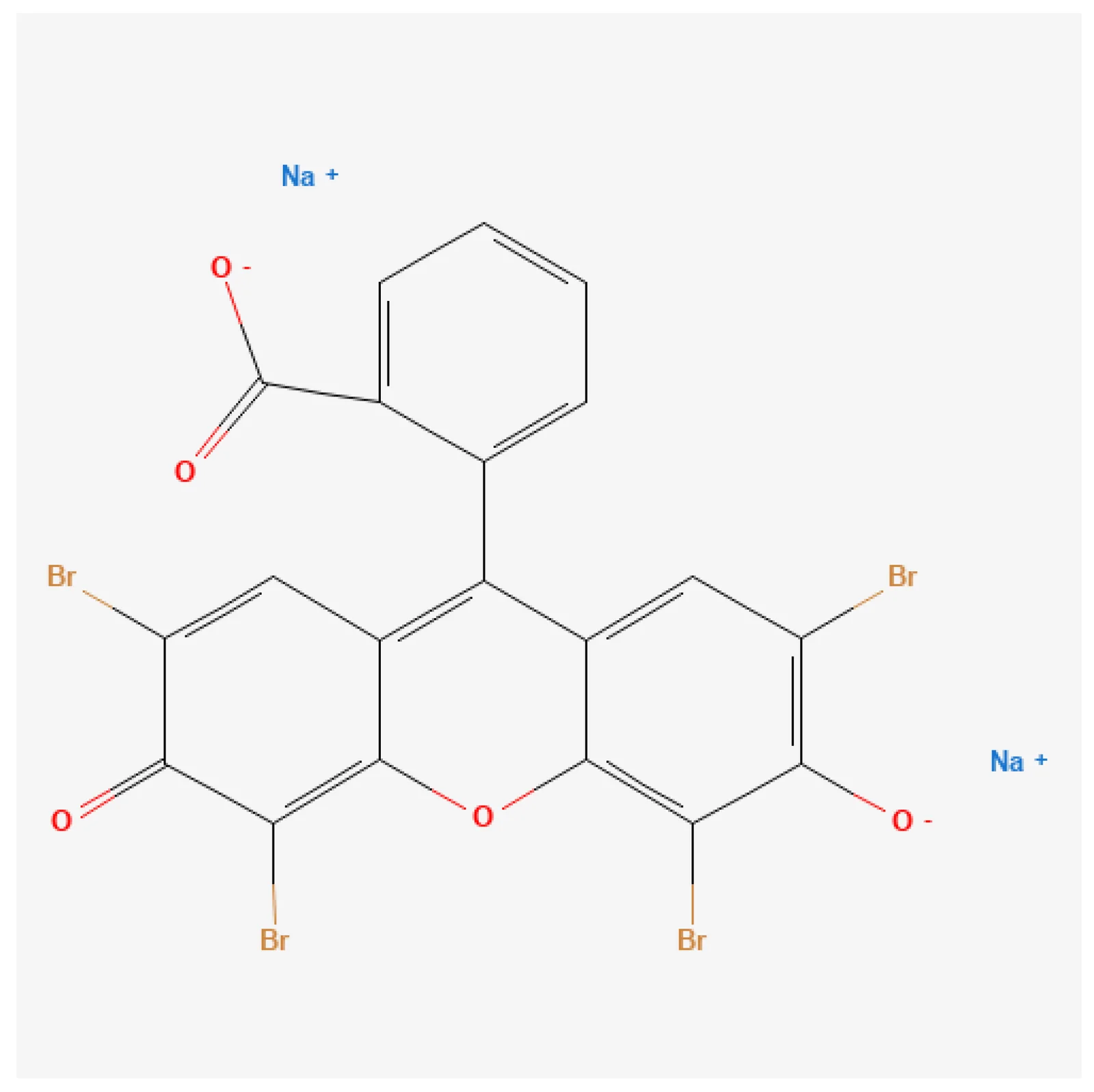
The photoinitiator Eosin Y.

A classic example of such a photoinitiator is eosin Y (Figure 4) (paired with triethanolamine, often in the presence of the co-monomer N-vinylpyrrolidone) and the camphorquinone/amine system. Unlike type I, here the radical is formed not by self-cleavage but through electron transfer: eosin Y operates via a photoinduced electron-transfer mechanism and requires a co-initiator (usually an amine). Upon excitation by light, eosin Y accepts an electron from the amine, producing a radical anion and a radical cation, the latter of which yields the initiating radical [18]. The key advantage of type II initiators is that they operate under visible light. Eosin Y is an FDA-approved dye that, paired with triethanolamine, initiates photopolymerization under visible light [58]. This resolves the problem of UV phototoxicity but entails a more complex formulation (a co-initiator and often a co-monomer) and more complex reaction kinetics.

#### 5.1.3. Metal-Containing (Ruthenium) Complexes

**Figure 5:**
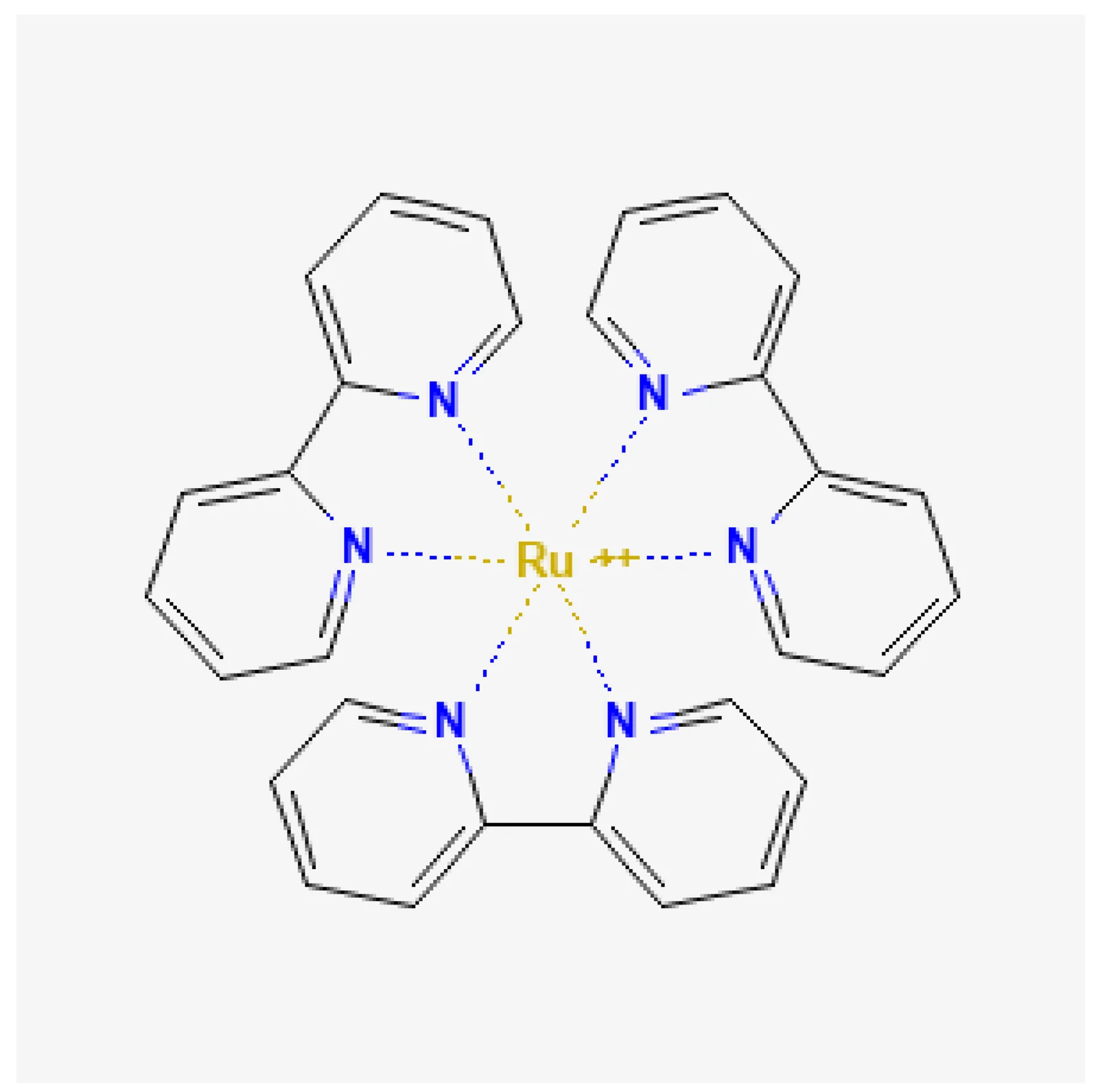
The photoinitiator Ru(bpy)_3_^2+^.

The ruthenium-based system Ru(bpy)_3_^2+^ (Figure 5) in the presence of sodium persulfate (SPS) constitutes a visible-light photoredox system. Under blue light (400–450 nm), Ru^2+^ is oxidized to Ru^3+^, donating an electron to SPS; the SPS that accepts the electron decomposes into sulfate anions and sulfate radicals, which then form covalent bonds between neighboring tyrosine residues or acrylate groups. The system is reported to be safe for corneal cells and fibroblasts [59]. Moreover, in the rate and efficiency of GelMA photopolymerization, the ruthenium system outperforms those based on I-2959 and LAP [58]. An important caveat should be made, however: the intrinsic biocompatibility of ruthenium has not been firmly established, and under certain conditions signs of high toxicity may appear [60].

#### 5.1.4. Nature-Inspired (Bioinspired) Initiators

**Figure 6:**
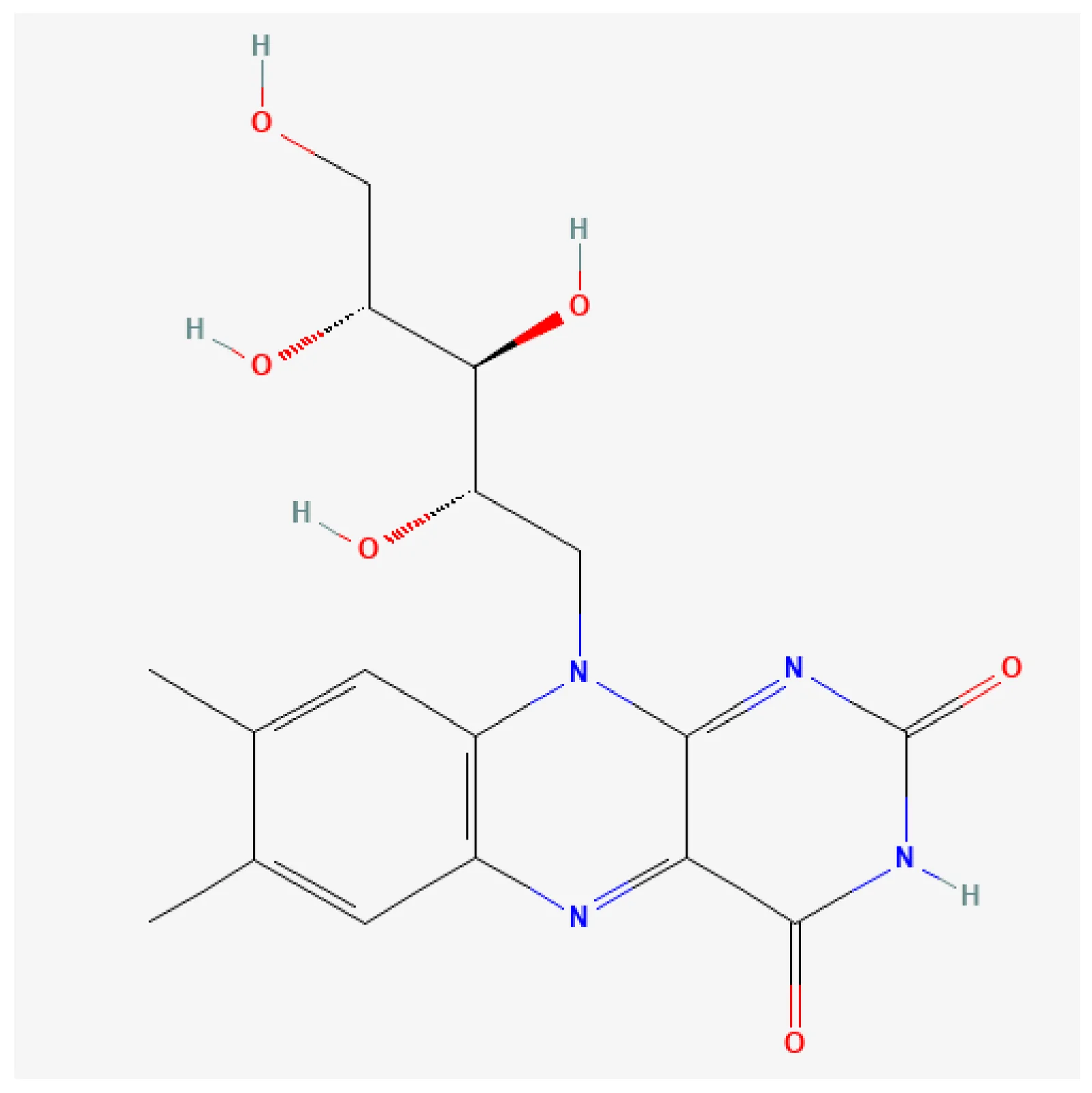
The photoinitiator riboflavin (vitamin B_2_).

Here, the role of photosensitizer is played by a natural molecule. Riboflavin (vitamin B_2_) is a non-toxic photoinitiator used to form gels under visible light (Figure 6). Its main drawback is slow gelation kinetics, which can, however, be eliminated by adding an electron acceptor (for example, sodium persulfate), providing fast, cytocompatible crosslinking [60]. The principal merit of this group is its established biocompatibility (a vitamin), which favorably distinguishes riboflavin from, for example, ruthenium complexes. For this reason, riboflavin paired with an electron acceptor is regarded as a bioinspired alternative to conventional type II systems. The therapeutic use of riboflavin at high doses or in combination with UV irradiation has already demonstrated compatibility with living organisms [61].

### 5.2. Influence of the Photoinitiator on System Properties

The choice of photoinitiator determines not only whether the reaction is triggered but also three interrelated groups of characteristics: gelation kinetics (rate, depth, and required irradiation dose), the mechanical properties of the final product (elastic modulus, porosity, resistance to degradation), and the cytotoxicity of the system. Each of these aspects is considered in turn below.

#### 5.2.1. Gelation Kinetics: Time, Power, and Depth

The rate of gelation is determined directly by the rate of radical generation, which in turn depends on initiator concentration, light intensity, quantum yield, and the fraction of absorbed radiation [19]. In practice, this means that varying the initiator concentration and source power can substantially affect crosslinking efficiency, assessed, for example, by the storage modulus [18]. Different types of initiators yield markedly different kinetics. In one comparative study, thin films (0.5 mm) could be obtained with a type I initiator only under relatively prolonged irradiation (4–5 min), whereas a type II initiator crosslinked thick (centimeter-scale) specimens in less than 60 s [62]. Kinetics can be further accelerated by adding co-monomers (for example, NVP or VC) [12]. The problem of oxygen inhibition, characteristic of acrylate systems, deserves separate attention. Oxygen slows crosslinking near the gel surface, and this effect must be taken into account when selecting the irradiation dose (the product of intensity and time).

#### 5.2.2. Mechanical Properties, Viscoelasticity, and Porosity

The dependence of gel stiffness on initiator concentration is not monotonic but passes through a maximum. For eosin Y, maximal stiffness is reached at a concentration of about 0.02 mM, whereas further increases in concentration reduce stiffness. For the ruthenium system, the upper limit was 0.5 mM [58]. Stiffer gels generally exhibit greater resistance to enzymatic degradation and less swelling. Differences between initiator types are also substantial. The mechanical properties of gels obtained with a type II initiator (eosin Y) were substantially—by up to 300—higher than those of gels made with a type I initiator [62]. For drug delivery, this implies a direct relationship: a higher crosslinking density (and hence a higher elastic modulus) reduces pore size and, consequently, slows diffusional drug release. In other words, the choice of initiator and its concentration simultaneously determines stiffness, porosity, and release kinetics.

Recently, it has become clear that stiffness alone does not capture the mechanical cues that matter to cells. Native extracellular matrices are viscoelastic and exhibit stress relaxation—a time-dependent response to deformation—whereas covalently crosslinked synthetic hydrogels are typically elastic [63,64]. This distinction is functionally important: matrix viscoelasticity and stress relaxation regulate fundamental cell processes, including spreading, proliferation, migration, and differentiation, and can promote behaviors not observed in purely elastic gels [63]. In a seminal study, faster stress relaxation enhanced the spreading, proliferation, and osteogenic differentiation of mesenchymal stem cells, independently of the initial modulus, degradation, and adhesion-ligand density [64], highlighting the relevance of viscoelasticity to tissue regeneration [63]. Time-dependent mechanics are equally consequential for delivery: the viscoelastic rheological properties of injectable hydrogels—shear thinning, yield stress, thixotropy, and self-healing—serve as quantitative indicators of injectability and controlled release [65]. Otherwise-elastic photocrosslinked networks can be endowed with such behavior by incorporating dynamic, reversible bonds (e.g., the Schiff-base/hydrazone chemistries) [34].

#### 5.2.3. Cytotoxicity: Mechanisms, Thresholds, and Minimization Strategies

The main mechanism of photoinitiator toxicity is associated with oxidative stress. Initiators generate reactive oxygen species (ROS): singlet oxygen, superoxide anion, hydroxyl radical, and hydrogen peroxide. Excessive ROS production damages cells. For type I initiators, it has been shown that an increase in elastic modulus is accompanied by increased ROS generation and a proportional decrease in cell viability [19]. Studies demonstrate that toxicity in this case is concentration-dependent. High concentrations of eosin Y (0.25–0.5%) lead to more pronounced cell death, with the reduction in viability related not only to ROS but also to a decrease in pore size, which impairs the mass transfer of nutrients [19]. As a formal benchmark, viability below 70% is considered indicative of cytotoxic potential according to international biocompatibility standards. Additional risks are associated with unreacted monomer (a known cytotoxicant) and lithium salts (which can cause acute kidney injury) [66].

Strategies for minimizing cytotoxicity include the following approaches:

- Reducing initiator loading to the minimal effective concentration. A system with minimal initiator loading has been shown to yield simultaneously a lower modulus, smaller pore size, and lower ROS levels, which markedly improves cell viability [19].
- Switching to visible-light initiators (LAP, eosin Y, ruthenium) or natural ones (riboflavin), which avoids UV irradiation.
- Washing out residual monomer and initiator after gel formation.
- Optimizing the irradiation dose without exceeding the necessary minimum.

#### 5.2.4. Polymerization Depth and Spatial Control

The depth at which a gel can form is limited by light attenuation. According to the Beer–Lambert–Bouguer law, incident light is absorbed predominantly in the upper layer, creating a gradient of initiating species and, consequently, a sharp cure-depth profile. Layers ranging from a few to several hundred micrometers are typically photopolymerized; the maximum achievable depth is a few millimeters [67]. Depth can be controlled in several ways. According to the same Beer–Lambert law, greater penetration depth is achieved by lowering the initiator concentration or by choosing an initiator with low molar absorptivity at the wavelength used [68]. Acting in the same direction are an increased irradiation dose and a shift to longer wavelengths (where light scattering in tissue is lower). Spatial control is provided by directed illumination (for example, through photomasks or projection systems). The highest resolution is afforded by two-photon polymerization, which allows three-dimensional structures to be formed with sub-micron precision (see Section 3.4). The choice of photoinitiator is thus always associated with the need for a trade-off. Accelerating surface crosslinking often limits polymerization depth, and vice versa. The optimal choice therefore depends on the specific task—whether a thin surface film or a bulk gel at depth is required.

Table 3 systematizes the key characteristics of the most common photoinitiators used for the photopolymerization of in situ hydrogels, including mechanism of action, activation wavelength, solubility, cytotoxicity, polymer compatibility, and regulatory status.

It is important to emphasize that none of the listed initiators has FDA/EMA approval specifically as a crosslinking photoinitiator for in situ delivery systems. The existing approvals pertain to other roles. Eosin Y is approved as a dye [58]. Riboflavin is on the FDA list of approved food colorants [73] and is also used clinically with UV-A for corneal crosslinking. In contrast, I-2959, LAP, and the ruthenium system remain at the research stage. For context, it is worth recalling that light-activatable agents do in principle reach the clinic: verteporfin (Visudyne) has been FDA-approved since 2000 as a light-activatable photosensitizer for photodynamic therapy of age-related macular degeneration [74]—but this is a therapeutic photosensitizer, not a gelation initiator, and the two should not be conflated. The transition of photo-responsive in situ systems into the clinic is thus hindered, in particular, by the absence of specifically approved initiators—which is currently a significant limitation.

### 5.3. Comparative Assessment and Selection Criteria

No single photoinitiator is universally optimal. The choice is a trade-off among activation wavelength, water solubility, cure speed, cytocompatibility, and regulatory status. The weighting of these factors depends on the intended application.

Among type I initiators, LAP has become the de facto standard for cell-laden systems. Its advantages over I-2959 are well defined. I-2959 absorbs in the UV, which is poorly matched to cell-compatible wavelengths. LAP, by contrast, is water-soluble and absorbs at both 365 and 405 nm. It also reaches gelation about ten times faster than I-2959 at 365 nm. More importantly, LAP can cure under cytocompatible 405 nm violet light at low concentration and low intensity. This regime is precluded for I-2959 by its absorbance profile. Under these conditions, encapsulated-cell survival exceeds 95% [71]. Single-component operation, visible-light compatibility, and rapid cure at low loading together explain its wide adoption. LAP is not free of liabilities. Photoexcitation generates reactive oxygen species. The combination of LAP and light, rather than LAP alone, is cytotoxic to mammalian cells. Its toxicity should therefore be assessed under actual irradiation rather than from extracts [72].

The visible-light alternatives are preferable when UV must be avoided altogether. Eosin Y and riboflavin both cure under visible light. They also carry regulatory familiarity, as an approved dye and a vitamin, respectively. The ruthenium/persulfate system offers the fastest visible cure and efficient tyrosine crosslinking. However, it is constrained by the unestablished intrinsic biocompatibility of ruthenium.

These properties translate into simple selection rules. LAP is the default for rapid, cytocompatible violet-light gelation. Eosin Y or riboflavin are chosen when a fully UV-free and regulatorily familiar system is required. Ruthenium is reserved for maximal-speed or tyrosine-based crosslinking, where its metal content is acceptable.

The translational outlook also differs by initiator. The clearest paths belong to riboflavin and eosin Y, given their existing clinical and regulatory familiarity. LAP will instead require a dedicated safety dossier that addresses its light-dependent cytotoxicity [72]. The broader gap is the absence of a defined regulatory pathway for photoinitiators as functional excipients. As a result, residual-initiator limits and light-dose specifications, set within the Quality by Design framework (Section 7), will be as decisive for approval as the chemistry itself.

## 6. Clinical Translation of Photo-Responsive Systems

Photocurable and photo-responsive materials have been used in experimental and clinical medicine for several decades. The largest body of data has accumulated in fields where the target is optically accessible and light irradiation is already integrated into standard treatment protocols: dentistry, ophthalmology, and regenerative medicine. In oncology, by contrast, the use of photo-responsive systems is limited to preclinical studies, although this direction is developing actively, drawing on clinical experience with photodynamic and photothermal therapy. This section considers the key application areas of photocurable in situ systems for which the most representative in vivo data have been accumulated, including both experimental animal models and clinical observations.

### 6.1. Dentistry

Light curing in dentistry has been a clinical routine for nearly five decades. Composite restorative materials, cured with blue-spectrum radiation (λ ≈ 468 nm), contain camphorquinone (type II) as the photoinitiator, functioning together with an amine co-initiator. Camphorquinone is recognized as a baseline photoinitiator in the recommendations of the American Dental Association (ADA) and in industry ISO standards [75]. This fact has substantial translational significance: the light-curing infrastructure and the photoinitiator itself have many years of clinical use and regulatory recognition, which lowers the barriers to introducing therapeutic photocurable depots into dental practice. The key preclinical applications of photocrosslinkable systems in dentistry are considered below.

#### 6.1.1. Periodontitis

One of the most illustrative examples is the composite hydrogel ZIF-8/GelMA (GelMA-Z) [76]. Zeolitic imidazolate frameworks (ZIFs) are a subclass of metal–organic coordination polymers that combine high porosity, loading capacity, biocompatibility, and pH-responsive degradation, which underlies their promise for targeted delivery [77]. Within GelMA-Z, ZIF-8 nanoparticles (a coordination polymer based on Zn^2+^ ions and 2-methylimidazole) serve as a reservoir and a pH-controlled source of zinc ions. In the acidic environment of an inflamed periodontal pocket, the framework degrades, releasing Zn^2+^ directly at the site of infection. The system thus implements two-level control: photoinduced formation and fixation of the depot in situ, together with stimulus-dependent release of the active agent. The liquid prepolymer formulation is injected into the periodontal pocket, fills its irregular cavity, and undergoes UV curing directly in place. In vitro, GelMA-Z increased the expression of osteogenic markers, elevated alkaline phosphatase activity, and stimulated mineralization of the extracellular matrix by rat bone-marrow mesenchymal stem cells, while showing pronounced antibacterial activity against *Porphyromonas gingivalis*—a key periodontitis pathogen. In a rat periodontitis model in vivo, the depot reduced bacterial load, attenuated the inflammatory response, and stimulated alveolar bone regeneration, with confirmed biocompatibility [76].

#### 6.1.2. Endodontics

In endodontic practice, photocrosslinking is used to localize aggressive disinfectants, in particular sodium hypochlorite (NaOCl), which in free form can extrude beyond the apical foramen and damage periapical tissues. Dal-Fabbro et al. developed an injectable GelMA hydrogel containing halloysite aluminosilicate nanotubes loaded with NaOCl [78]. The nanotubes serve as a reservoir, while the photocrosslinked GelMA matrix provides retention and sustained release of the disinfectant. The material exhibited a stable pore size (70–80 µm), a compressive modulus of 100–200 kPa (independent of loading), a swelling ratio of 6–7, and complete degradation by day 7; cytocompatibility recovered to 65–80% after an initial decline. The optimal composition provided the largest zone of microbial growth inhibition and pronounced biofilm disruption. In a rat subcutaneous model at 21 days, the gel had degraded only partially, causing moderate local inflammation without tissue necrosis [78]. This example demonstrates that a photocurable matrix can safely deliver a substance that in free form is damaging to surrounding tissues.

#### 6.1.3. Maxillofacial Surgery and Hemostasis

Photocrosslinking also finds use as a tool for local hemostasis. Chang et al. proposed an injectable bioadhesive based on GelMA and gelatin modified with phenyl isothiocyanate (Gel-Phe), in which photocrosslinking is complemented by supramolecular interactions, and hemostatic activity arises from phenyl-isothiourea and amino groups that accelerate blood adsorption and clotting [79]. Varying the molar ratio of gelatin to phenyl isothiocyanate (from 1:1 to 1:15) and the UV irradiation time (15–60 s) allowed injectability and mechanical properties to be tuned. The optimal composition (10% GelMA/10% Gel-Phe 1:15) was applied directly to the bleeding surface and cured in 60 s. In ex vivo bleeding models (porcine skin, a socket of an extracted tooth), the adhesive reduced clotting time by 45% with low cytotoxicity, and after clot formation it was easily removed without re-traumatizing the wound [79].

Dentistry thus has a pronounced translational advantage for photocurable delivery systems, owing to the already-existing clinical infrastructure of light curing and to regulatorily recognized photoinitiators, which makes it possible to use standard dental equipment to polymerize therapeutic depots. At the same time, it should be noted that dedicated clinical trials of photocurable drug-delivery systems in dentistry have not been registered to date, and all the examples presented are at the preclinical stage of development.

Table 4 summarizes the key examples of photocurable systems in dentistry—from the treatment of periodontitis and endodontics to local hemostasis.

### 6.2. Ophthalmology

The optical accessibility of the eye and the acute clinical need for sustained drug delivery to the posterior segment (age-related macular degeneration, diabetic retinopathy) make ophthalmology one of the most promising niches for photocurable in situ systems [80]. An intravitreal depot formed in situ can reduce the frequency of injections and the associated complications.

#### 6.2.1. Posterior Segment: Retinal Delivery and Vitreous Substitutes

The main scenario envisions creating a depot that provides prolonged release of a therapeutic agent. An intravitreal injectable GelMA-based system containing triamcinolone acetonide showed low toxicity, controllable swelling, high biocompatibility, and more prolonged release than a suspension formulation; upon implantation into rabbit eyes, all retinal layers retained normal function [81]. In addition, photocurable hydrogels based on hyaluronic acid and GelMA double networks are being developed as vitreous substitutes capable of restoring intraocular pressure and maintaining the shape of the eye [81]. The photocurable in situ platform most advanced toward clinical use is the Re-Vana Therapeutics technology (the OcuLief variant). The formulation is administered through a fine needle (23–27 G) and photocured directly in the vitreous body using a pulsed visible-light source without significant thermal effects, which preserves high-molecular-weight biopharmaceuticals and allows the procedure to be performed on an outpatient basis. By adjusting crosslinking density, the release rate can be tuned within the range of 0.05–20 µg/day over a period of 6 to 12 months [82]. In July 2025, Re-Vana entered into a strategic licensing agreement with Boehringer Ingelheim to develop sustained-release ophthalmic products; the value of the deal for the first three programs exceeds US$1 billion [83]. However, public clinical data on this platform were not available at the time of preparing this review.

#### 6.2.2. Anterior Segment: Cornea and Ocular Surface

For the treatment of inflammatory diseases of the ocular surface, a subconjunctival gel based on a G4-PAMAM dendrimer, hyaluronic acid, and dexamethasone has been developed. After photocuring, the bioavailability of dexamethasone increased and release became sustained; in a rat corneal alkali-burn model, the gel significantly reduced corneal thickness, neovascularization, and the level of inflammatory cytokines compared with the free drug [81]. Transparent corneal fillers based on GelMA, cured with visible light (100 mW/cm^2^) using an eosin Y/triethanolamine/N-vinylcaprolactam photoinitiating system, have been developed, illustrating a practical implementation of the shift to visible-spectrum initiators [84]. The adhesive system OcuPair, an in situ photocurable composite of methacrylated PAMAM dendrimer and hyaluronic acid, forms a transparent, watertight film over full-thickness corneal defects within 90 s [85]. The authors note the absence of FDA-approved corneal adhesives, which underscores the persistent gap between preclinical developments and clinical implementation [85].

Corneal crosslinking (CXL) with riboflavin and UV-A radiation deserves separate mention; strictly speaking, it is not a drug-delivery system but represents the most clinically validated example of photochemical crosslinking of a biological tissue. According to the classic Dresden protocol (Wollensak et al., 2003), after mechanical removal of the central epithelium, the stroma is soaked with a 0.1% riboflavin solution for 30 min, followed by UV-A irradiation (370 nm, 3 mW/cm^2^, total dose 5.4 J/cm^2^) [86]. The reactive oxygen species generated initiate the formation of covalent crosslinks between collagen fibrils, which increases the mechanical strength of the cornea and halts the progression of keratoconus; in the first clinical study, in 23 eyes, disease stabilization was achieved in all patients [86]. The PACK-CXL (Photoactivated Chromophore for Infectious Keratitis) variant applies the same photochemical system to treat infectious keratitis, where the reactive oxygen species exert a direct antimicrobial effect and stromal crosslinking prevents its enzymatic melting [87].

Table 5 summarizes the key examples of photochemical crosslinking and photocurable systems in ophthalmology, including clinically approved protocols and preclinical developments.

In ophthalmology, therefore, a substantial body of preclinical data has accumulated, and there are successful clinical examples of photochemical action on tissues; however, photopolymerizable drug-delivery depots have not yet reached the stage of registration trials, with the exception of isolated platforms in the pivotal-study phase.

### 6.3. Regenerative Medicine

In regenerative medicine, photocurable hydrogels have the longest clinical history, but predominantly as surgical sealants and tissue-engineering constructs rather than as drug-delivery systems. Notably, the photoinitiators used in these products belong to the same classes as those considered above. The earliest and most illustrative example is the sealant FocalSeal-L, approved by the FDA in 2000 for sealing air leaks after lung resection. The material is a PEG-containing macromer with acrylate end groups (a copolymer of PEG with trimethylene carbonate and lactide). Curing is initiated by an eosin Y-based primer under blue-green light. The formulation is applied in two steps; hydrolytic degradation of the gel in vivo is completed within a few weeks [88]. In a multicenter randomized study, FocalSeal-L achieved control of air leaks in 92% of treated patients; by the time of discharge, the proportion of patients without a leak was 39% versus 11% in the control group (*p* ≤ 0.001) [89]. Reported drawbacks were the two-step application, the requirement for a dry surgical field, and a slight increase in the rate of infectious complications [88].

A second significant example is the GelrinC implant for the repair of articular knee cartilage. GelrinC is a composition of PEG diacrylate and denatured fibrinogen (the PEG-fibrinogen platform). The material is introduced into the cartilage defect in liquid form after microfracture and undergoes UV curing in situ over 90 s, forming a soft, elastic, acellular scaffold. Over 6–12 months, the implant is resorbed and replaced with hyaline-like tissue. A pivotal study is currently under way within an FDA IDE (comparison with microfracture, NCT03262909) [90]. In prospective observations with MRI evaluation (MOCART, T2 mapping), improvement in the quality of the regenerate was noted up to 24 months [91]. GelrinC represents a rare example of an in situ photopolymerizable implant that has achieved regulatory approval and pivotal trials in orthopedics.

To these two clinically validated products may be added corneal crosslinking (Photrexa/KXL, FDA, 2016), already considered in Section 6.2, as well as the overwhelming majority of preclinical developments carried out with GelMA and the photoinitiators LAP or Irgacure 2959 for the engineering of cartilage, bone, vasculature, and skin. To date, none of these platforms has been brought to market as a commercial product for tissue regeneration [12]. Table 6 presents clinically validated and preclinical examples of photocurable hydrogels in regenerative medicine, including sealants (FocalSeal-L), implants (GelrinC), and GelMA-based tissue-engineering scaffolds.

Regenerative medicine thus convincingly demonstrates the clinical viability of photopolymerization and the corresponding photoinitiators in structural and sealing applications, setting a precedent for transferring this technology to the field of controlled drug release.

### 6.4. Oncology

The use of in situ forming systems in oncology is driven by the clinical need for local delivery of therapeutic agents to the tumor-resection site in order to suppress residual tumor tissue and reduce the risk of postoperative recurrence [92]. Photocurable hydrogels offer a solution that makes it possible to fill irregularly shaped post-resection cavities with subsequent in situ fixation of the depot and controlled release of cytostatic agents. This section considers two main approaches: local photocurable depots for chemotherapy and systems that combine chemotherapy with photothermal therapy activated by near-infrared radiation.

#### 6.4.1. Local Photocurable Depots for Chemotherapy

In a classic study, an aqueous solution of photocurable chitosan bearing azide and lactose groups (Az-CH-LA), containing paclitaxel, was subjected to UV irradiation (30 s) to form an insoluble gel. Paclitaxel retained its biological activity for at least 21 days and was released as the polymer matrix underwent enzymatic degradation. In a subcutaneous Lewis lung tumor (3LL) model in mice, the depot suppressed tumor growth and reduced the number of CD34-positive vessels, indicating inhibition of angiogenesis; antitumor efficacy exceeded that of free paclitaxel and of the empty gel [93]. In subsequent studies, the principle was adapted for activation by visible light: a gel based on glycol chitosan with a paclitaxel/β-cyclodextrin complex cured within 10 s of irradiation and provided controlled drug release over 7 days in an ovarian cancer model in mice [94]. An example of a system with tunable properties is provided by acrylated PEG-PCL macromers. A PEG-PCL triblock copolymer with terminal acrylate groups (PEG-PCL-Acr) is mixed with acrylated PEG or monomethoxy-PEG and cured with visible light in the presence of LAP to form a porous, interconnected network. Swelling and the rate of enzymatic degradation are determined by the composition; doxorubicin was used as the model antitumor agent [10]. An alternative approach that does not require photoinitiation is represented by a fibrin gel formed in the resection cavity from thrombin and fibrinogen with encapsulated doxorubicin. In this system, nitric oxide release was additionally activated by NIR irradiation (808 nm), which suppressed postoperative recurrence [95].

#### 6.4.2. NIR-Activated Chemo-Photothermal Therapy

In this approach, light controls not the formation of the gel but the release of the drug from a pre-formed depot through a photothermal mechanism. Nanocomposite gels with gold nanorods exhibit NIR and pH sensitivity, providing doxorubicin release over more than a month and realizing a combined chemo-photothermal therapeutic effect [54]. Analogous platforms based on hyaluronic acid with gold nanorods provide NIR-controlled release through local heating induced by surface plasmon resonance [96]. A photocatalytic CO-releasing sprayable hydrogel has been developed based on sodium alginate (ionic crosslinking with Ca^2+^) incorporating a g-C_3_N_4_ nanophotocatalyst modified with gold nanoparticles (C_3_N_4_/Au). Under visible light, the catalyst converts CO_2_ into carbon monoxide directly within the tumor tissue; combination with folic-acid-conjugated doxorubicin micelles enhances the antitumor effect and promotes the elimination of residual cells in breast cancer [87]. The hydrogel BP@PLEL, containing black phosphorus nanosheets in a thermoresponsive PDLLA-PEG-PDLLA matrix, undergoes a rapid sol–gel transition when applied to a postoperative wound and irradiated with NIR, and is used for local ablation of residual tumor tissue [55]. Composite systems that combine depot formation and light-controlled release within a single platform are of interest. In a thermoresponsive PLGA-PEG-PLGA matrix with doxorubicin nanoparticles (DOX@polydopamine@P (NIPAAm-co-AM), DPN), gelation occurs at physiological temperature; under NIR irradiation, polydopamine generates heat, providing controlled release of up to 87% of the drug while maintaining baseline release in the absence of irradiation. In a tumor-growth model, suppression reached 98.77% [97]. A key feature of such systems is the combination of two therapeutic modes within a single depot: NIR irradiation simultaneously initiates release of the chemotherapeutic agent and provides a photothermal effect on tumor cells.

Table 7 summarizes the key preclinical examples of photo-responsive in situ hydrogels in oncology, including local chemotherapeutic depots and systems for NIR-activated chemo-photothermal therapy.

Despite a substantial body of preclinical data, no photo-responsive in situ drug-delivery system has reached the clinical-trial stage in oncology. Notably, even those in situ gels that are undergoing clinical evaluation (for example, UGN-102 for intravesical chemotherapy of bladder cancer) are thermoresponsive rather than photocurable systems [7]. The in situ gel platform has thus demonstrated clinical viability, yet the photoactivated triggering mechanism remains at the preclinical level, which points to unresolved problems in biosafety, activation efficiency, and regulatory recognition.

## 7. Standardization and Quality by Design

### 7.1. The Problem of Standardization as a Barrier to Translation

The very flexibility that makes photocrosslinkable systems attractive is also the root of the reproducibility problem. The properties of such hydrogels depend strongly on the conditions of synthesis and processing, and the lack of standardization leads to conflicting results—this has been shown even for the most common material, GelMA, for which a unified standard had to be specifically proposed, defining the density of the molecular network through measurable parameters—the degree of substitution and the dry-matter fraction [99]. To this materials-science variability is added variability in light: the key photopolymerization parameters—the type and concentration of initiator, light intensity, and irradiation time—significantly affect the properties of the resulting gel [58]. Moreover, a systematic comparison was long lacking: the first systematic comparison of even three common initiators (eosin Y, LAP, ruthenium) on GelMA at a single irradiation intensity was carried out only in 2025 [58]. The consequence for the entire field is clear: because different groups use different light sources, intensities, doses, and initiator concentrations, and apply different characterization methods, their results are mutually incomparable and difficult to reproduce. This is precisely the third barrier to translation—alongside the absence of approved initiators (Section 5.3) and the limited depth of light penetration (Section 3.4): without reproducible and comparable characterization, a system cannot pass regulatory evaluation. Standardization here is therefore a precondition for reproducibility and clinical translation.

### 7.2. The Quality by Design (QbD) Concept

A systematic answer to the reproducibility problem is provided by the Quality by Design (QbD) concept, established in the ICH Q8–Q10 guidelines. In contrast to an empirical trial-and-error approach, QbD begins with predefined objectives and emphasizes understanding and controlling the product and process on the basis of scientific data and risk analysis [100]. The logic proceeds sequentially. First, a quality target product profile (QTPP) is formulated—a summary of the desired characteristics of the finished product. From it are derived the critical quality attributes (CQAs)—those physical, chemical, and biological properties that must remain within defined limits. Next, the factors influencing these attributes are identified, namely the critical material attributes (CMAs) and critical process parameters (CPPs). Finally, on the basis of risk assessment, a control strategy is built and the design space is delineated—that is, the range of parameters within which product quality is assured [100]. This framework is especially apt for photo-responsive in situ systems. QbD is already applied to various delivery systems, including in situ and injectable gels [100]; however, photosystems have a fundamental peculiarity: the final crosslinking occurs at the moment of application, “at the bedside,” so that the act of irradiation itself—wavelength, intensity, dose—becomes a critical process parameter performed outside the factory. This raises the stakes: the product is partly “manufactured” directly in the patient, and for this reason the rigorous definition of the QTPP, CQAs, and CPPs for such systems is no less—indeed, even more—important than for conventional dosage forms.

### 7.3. Critical Process Parameters (CPPs): The Specifics of Light

The distinctive feature of photosystems is that irradiation itself introduces a set of interacting critical process parameters. Their individual effects were described in Section 3.4 and Section 5 and are only summarized here. The wavelength must match the absorption of the chosen initiator. It also sets a trade-off between cytotoxicity and penetration depth, which is why a shift toward visible light or NIR (via upconverting nanoparticles) is used to reach greater depth [16,58]. Intensity accelerates radical generation and crosslinking [19]. The dependence is nonlinear, because higher intensity also depletes the initiator and dissolved oxygen and raises the thermal and oxidative load [67]. The integral dose, the product of intensity and time, determines the degree of double-bond conversion and the completeness of crosslinking [67]. Here too there is a trade-off, since a larger dose generates more reactive oxygen species and photodamage [19].

The type and concentration of initiator lie at the boundary between material and process. They set the rate of radical formation and, through it, the kinetics, mechanics, residual substances, and ROS level. Together with intensity, initiator concentration determines crosslinking efficiency, as measured by the modulus [18]. Each initiator has its own thresholds and plateaus, beyond which the gel behavior changes qualitatively [58]. Oxygen scavenges radicals and inhibits crosslinking at the surface, creating conversion and network-density gradients with depth. This inhibition is reduced by raising the initiator concentration or the dose, or by working in an oxygen-free environment, but in vivo it remains poorly controllable [67]. Cure depth follows the Beer–Lambert law and is managed through the initiator concentration and its molar absorptivity at the working wavelength [67,68]. Finally, the dose reaching the gel is not the dose at the source, because tissue scatters and absorbs light [16].

The practical core of QbD for photosystems follows from this coupling. These parameters must be optimized jointly and reported in full—wavelength, intensity, time, dose, initiator type and concentration, source type, distance, and spot size—so that studies become comparable.

### 7.4. Critical Quality Attributes (CQAs) of Photo-Responsive In Situ Delivery Systems and Their Characterization Methods

The critical process parameters (CPPs) that determine the success of photocuring were considered above. However, for the purposes of quality control and regulatory recognition, it is necessary to formalize the final characteristics of the product. Within the QbD methodology, these characteristics are formulated as critical quality attributes (CQAs)—physical, chemical, and biological properties that must remain within defined limits to ensure the safety and efficacy of the system. Table 8 lists the key CQAs for photo-responsive in situ hydrogels, the recommended methods for their quantitative assessment, and the corresponding critical process parameters that influence each attribute. This list is not exhaustive and may be adapted depending on the specific formulation and clinical application; nevertheless, the proposed system of criteria represents a minimal basis for the reproducible characterization and comparative analysis of such systems in preclinical studies.

### 7.5. Risk Assessment

**Figure 7:**
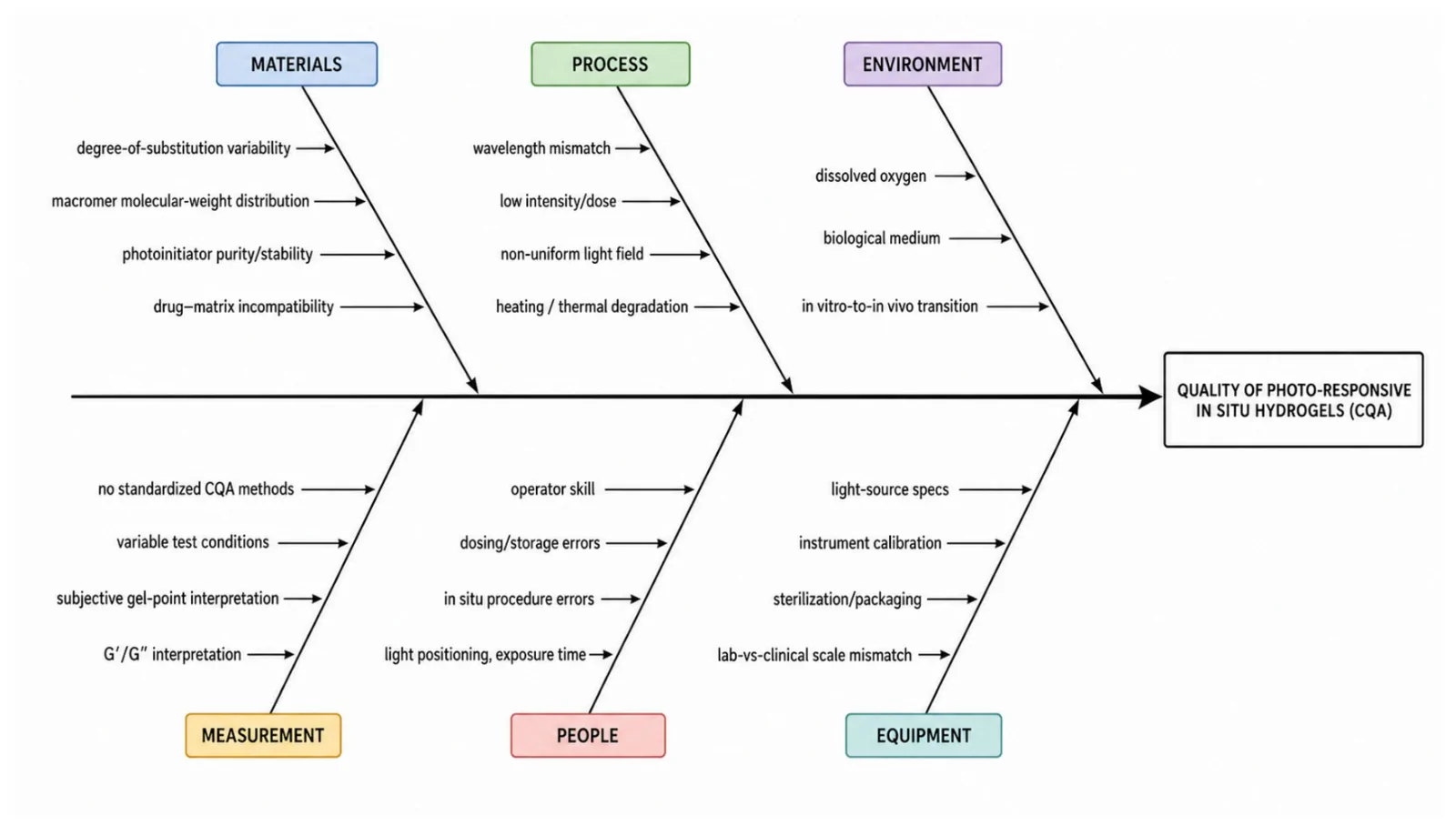
Ishikawa diagram of the risk factors affecting the quality of photo-responsive in situ hydrogels for drug delivery.

An integral element of the Quality by Design concept is a formalized risk assessment aimed at identifying factors capable of critically influencing the quality of the final product [100]. In accordance with the ICH Q9 guideline, risk assessment makes it possible to systematize the relationships among critical process parameters (CPPs), critical material attributes (CMAs), and critical quality attributes (CQAs), and to identify priority directions for experimental validation and control strategies. For photo-responsive in situ hydrogels, the risks are multifactorial in nature, encompassing both aspects classic for pharmaceutical development (polymer stability, reproducibility of synthesis, sterility) and specific factors associated with photochemical activation. Figure 7 presents an Ishikawa (“fishbone”) diagram that systematizes the main categories of risk affecting the quality of a photocurable in situ system.

### 7.6. Regulatory Framework and Reporting Requirements

A photo-responsive in situ gel containing a drug almost always turns out to be a combination product: it unites a medicinal substance with a device (the gel-forming material) and often with a third component—the light source. Both the FDA and the EMA regard such items as drug–device combinations and classify the hydrogel itself as a high-risk device: in the EU, under Regulation MDR 2017/745, this is class IIb or III; in China, class III—which entails the longest and most extensive preclinical and clinical testing [102]. In the United States, hydrogels fall under the definition of a device (section 201(g) of the FD&C Act), but the encapsulation of a drug turns them into a combination product, whose registration can take from 7 to 12 years or more [103]. The very diversity of crosslinking polymers and chemistries makes the regulatory classification of such systems a nontrivial task [103]. For photosystems, two additional barriers are superimposed on this. First, the photoinitiator is generally not an approved excipient (Section 5.3): the introduction of a new component with a new mechanism of action requires a separate safety assessment, and its residual amounts and the reactive oxygen species it generates become explicit safety control points. Second, the in situ curing step itself—wavelength, dose, time, initiator concentration—must be specified and justified for the particular clinical application, since it is this step that forms the final product directly in the patient. Biological evaluation is performed according to the ISO 10993 series of standards, but the presence of the drug may itself affect its results, so the safety of the material and the drug must be assessed jointly [101]. A further difficulty is that, for photocrosslinkable in situ systems, there is as yet neither a dedicated standard nor a pharmacopeial monograph, and regulators cannot always provide detailed testing requirements for novel devices in advance—which makes early dialog with agencies and a QbD-based dossier especially valuable. A practical bridge here is complete and uniform reporting: each study should specify both the irradiation parameters (6.3) and the quality attributes together with the methods for their determination (6.4), so that results become comparable and a regulatory-grade evidence base gradually accumulates. Aligning development with the ICH Q8–Q10 guidelines and the ISO 10993 series [100], as well as using expedited regulatory pathways for areas of unmet need (for example, RMAT in the United States and PRIME in the EU), is the most realistic route from preclinical successes to approval.

## 8. Discussion

Several conclusions emerge from weighing the reviewed studies against one another. Based on current evidence, the most clinically advanced systems are not the most elaborate ones. The platforms closest to the clinic rely on simple, already-accepted photochemistry: riboflavin/UV-A crosslinking of collagen and PEG-based products such as FocalSeal-L and GelrinC (Section 6).

The most versatile platform for a drug-delivery depot, by contrast, is visible-light-cured methacryloyl gelatin. It combines bioactivity, tunable properties, and the largest preclinical record. Naturally derived polysaccharides such as methacrylated hyaluronic acid and gellan gum are most attractive where tissue-specific function or injectability is decisive, but they remain mechanically weaker and less tested.

This comparison reframes matrix design. The priority is no longer a universal material but the rational matching of matrix, crosslinking chemistry, and degradation profile to the target tissue. Alongside this, a few design choices are emerging as consistently advantageous: the shift from UV to visible-light initiation, the use of orthogonal or dual crosslinking to decouple mechanics from gelation, and the deliberate introduction of viscoelastic, stress-relaxing behavior (Section 4.2, Section 5.2.2 and Section 5.3).

More complex is the question of photoinitiator selection. For a number of compounds widely used in research, the regulatory status and safety profile under injectable administration remain undefined, which constrains clinical translation more than the limitations of the crosslinking chemistry itself. A rational strategy appears to be the use of already-approved and clinically validated initiators and photosensitizers—above all riboflavin, as well as camphorquinone and eosin Y—which reduces regulatory uncertainty and shifts the research focus to optimizing the delivery system proper.

The systematic application of a Quality by Design strategy should be regarded as the decisive condition for reproducibility and regulatory recognition. Accumulating empirical data that establish the relationship between critical process parameters (CPPs) and critical quality attributes (CQAs) will make it possible to move from a predominantly qualitative description of these elements to operating with their optimal quantitative values and, consequently, to an objective quantitative assessment of pharmaceutical-development risks. Progress in this field is thus determined not by awaiting a single breakthrough but by coordinated advancement along three directions: targeted engineering of matrices, reliance on clinically accepted photoinitiators, and a transition to quantitative, QbD-oriented characterization.

## 9. Conclusions

This review shows that photo-responsive in situ forming hydrogels are a flexible design platform in which light serves as a precise tool for spatiotemporal control. It is precisely their modularity—the free choice of polymer matrix, photoinitiator, and wavelength for a specific task—that accounts for the wealth of preclinical results accumulated in dentistry, ophthalmology, regenerative medicine, and oncology, and makes this direction one of the most promising in local drug delivery.

No photocrosslinkable injectable depot has yet been approved as such. However, the underlying photochemistry and several of these initiators are already clinically established in adjacent fields (Section 6 and Discussion). The task is therefore one of transferring a mature technology to a new role as a controlled-release depot.

The review also clearly outlines what is required for this, reducing the task to three manageable barriers: qualification of initiators specifically for injectable use, the barrier of light penetration into tissue, and standardization of characterization. The last point is of particular practical value: the Quality by Design approach proposed in this review, with mandatory documentation of irradiation parameters and quality attributes, is a concrete tool, within the community’s own reach, capable of rapidly turning scattered data into a cumulative evidence base.

## Figures and Tables

**Table 1:** Comparative characteristics of selected photo-responsive platforms: composition and crosslinking.

Matrix	Type	Crosslinking Chemistry	Photocuring Conditions	Key Biological Feature	Degradation	Tuning Parameter	Reference
GelMA	Natural	Methacrylate, chain-growth	UV/visible + photoinitiator; seconds–minutes	RGD motifs, MMP-cleavage sites	Enzymatic (MMP)	Degree of substitution	[12,29]
GelNB	Natural	Thiol–ene, step-growth	Visible/long-UV + thiol crosslinker; fast	Homogeneous network, low shrinkage	Enzymatic	Degree of substitution, thiol crosslinker	[30]
HAMA/MeHA	Natural	Methacrylate	UV/visible + photoinitiator	CD44 ligand (tumor targeting)	Enzymatic (hyaluronidase)	Degree of substitution	[25,31]
Glycol chitosan-MA	Natural	Methacrylate (dual: thermo + photo)	UV/visible + photoinitiator	Mucoadhesion, antibacterial	Enzymatic/physical	Degree of substitution, physical crosslinking	[35,36,37]
GGMA/MeGG	Natural	Methacrylate (photo/ionic/thermal)	UV/visible (ruthenium/persulfate); 30–120 s	GAG-like; orthogonal crosslinking	Enzymatic (lysozyme), methacrylation-dependent	Degree of methacrylation, cation content	[41,42]
Collagen (riboflavin)	Natural	ROS-mediated	Riboflavin·UV-A or rose bengal·green light	Native ECM microenvironment	Enzymatic, fast if uncrosslinked	Light/riboflavin dose	[28,38,40]
PEGDA	Synthetic	Acrylate, chain-growth	UV/visible + photoinitiator; seconds–90 s	Bioinertness, low protein adsorption	Hydrolytic (ester)	Molecular weight, concentration	[45,46,47]

**Table 2:** Comparative characteristics of selected photo-responsive platforms: functional and translational profile.

Matrix	Modulus (kPa)	Injectability	Drug Loading	Clinical Maturity	Advantages (+)/limitations (−)	Reference
GelMA	Tunable, ~1–200	Good	High	Preclinical (broadest)	+Bioactive, most versatile −O_2_ inhibition, batch variability	[12,29]
GelNB	Tunable	Good	High	Preclinical, early	+Uniform network, O_2_-insensitive −Needs thiol crosslinker	[30]
HAMA/MeHA	Low–moderate	Good	High	Preclinical	+CD44 tumor targeting −Weak; high methacrylation lowers targeting	[25,31]
Glycol chitosan-MA	Moderate	Good	Moderate–high	Preclinical	+Mucoadhesive, antibacterial −Insoluble at physiological pH	[35,36,37]
GGMA/MeGG	Tunable, ~6–17	High	Moderate	Preclinical (cartilage, bone)	+Orthogonal crosslinking, food-additive safety −Few adhesion motifs, needs cations	[41,42]
Collagen (riboflavin/RGX)	Low, raised by crosslinking	High	Moderate	Clinical (corneal crosslinking)	+Native ECM, clinically used −Low intrinsic stiffness	[28,38]
PEGDA	High, molecular-weight-tunable	Good	Moderate	Clinical (blends: FocalSeal, GelrinC)	+Defined, bioinert, precedented −No cell adhesion	[45,46,47]

**Table 3:** Comparative characteristics of photoinitiators.

Initiator (Type)	Activation Wavelength	Polymer Compatibility	Regulatory Status	Advantages (+)/Limitations (−)	Ref.
Irgacure 2959 (type I)	~280 nm (UV), weak at 365 nm	Acrylates/methacrylates (GelMA, PEGDA)	Industrial initiator, not clinically approved	+Long-established, no co-initiator needed −UV-only, phototoxic under prolonged irradiation, largely superseded by LAP	[69,70]
LAP (type I)	365 and 405 nm (violet/blue)	Acrylates/methacrylates, thiol–ene (collagen–PEG)	Research standard, not clinically approved	+Water-soluble, fast, cytocompatible 405 nm cure, single-component −Photo-ROS cytotoxic under light, lithium salt	[58,69,71,72]
Eosin Y (+TEA, type II)	Visible, ~515–525 nm	Acrylates/methacrylates (GelMA)	FDA-approved as a dye	+Visible light, approved dye −Needs co-initiator and co-monomer, cytotoxic at high concentration	[19,58]
Ru(bpy)_3_/SPS (metal complex)	Visible, 400–450 nm (blue)	Tyrosine (collagen, gelatin, silk) + acrylates	Research stage, not approved	+Fastest visible cure, tyrosine crosslinking −Ruthenium biocompatibility unproven	[59,60]
Riboflavin (+acceptor, natural)	Visible (~450 nm) and UV-A	Silk, collagen, methacrylated polymers	Vitamin/dye, FDA-approved; used clinically with UV-A (corneal crosslinking)	+Vitamin, established safety, visible light −Slow without an electron acceptor	[60,73]

**Table 4:** Photocrosslinkable systems in dentistry.

System	Light and Mechanism	Drug/Agent	Model and Outcome	Ref.
ZIF-8/GelMA (GelMA-Z), periodontitis	UV, in situ photopolymerization in the pocket; ZIF-8 adds pH-dependent release	Zn^2+^ ions	Rat periodontitis model: reduced bacterial load and inflammation, alveolar bone regeneration; in vitro—activity against P. gingivalis and osteogenesis	[76]
GelMA with halloysite nanotubes, endodontics	UV, photopolymerization; matrix retains and gradually releases the disinfectant	Sodium hypochlorite (NaOCl)	Pores 70–80 µm, modulus 100–200 kPa, complete degradation by day 7; antibacterial and antibiofilm effect; rat subcutaneous—moderate inflammation without necrosis	[78]
GelMA/Gel-Phe bioadhesive, hemostasis	UV 15–60 s; photocrosslinking plus supramolecular interactions	Hemostatic without a drug (phenyl-isothiourea and amino groups)	In vitro models (porcine skin, tooth socket): clotting time reduced by 45%, easily removed after clot formation	[79]

**Table 5:** Photocrosslinkable systems in ophthalmology.

System	Light and Mechanism	Drug/Agent	Model and Outcome	Ref.
Corneal crosslinking (CXL)	UV-A (370 nm, 3 mW/cm^2^, 5.4 J/cm^2^); riboflavin generates ROS that crosslink stromal collagen	Riboflavin (photosensitizer; crosslinks native tissue, not a depot)	Clinical standard for keratoconus; in the first study (23 eyes), progression halted in all	[86]
PACK-CXL	UV-A plus riboflavin (same principle)	Riboflavin	Infectious keratitis: direct microbial damage by ROS and protection of the stroma from enzymatic “melting”	[87]
GelCORE (GelMA corneal bioadhesive)	Visible light, eosin Y; in situ photocrosslinking	Sutureless corneal sealant (drug-free)	Ex vivo and in vivo (rabbit): sealing of corneal defects, structural restoration, re-epithelialization	[84]
OcuPair (PAMAM dendrimer/hyaluronic acid)	In situ photocrosslinking	Bioadhesive “bandage” for temporary corneal repair	Preclinical: adhesion and closure of corneal injuries	[85]
Re-Vana OcuLief (intravitreal implant)	Visible light; in situ photocrosslinking	Sustained-release depot	Closest to the clinic among photocrosslinkable ocular depots	[82]

**Table 6:** Clinically applied and translationally significant photopolymerizable systems in regenerative medicine.

System	Light and Mechanism	Drug/Agent	Model and Outcome	Ref.
FocalSeal-L (PEG-co-trimethylene carbonate-co-lactide with acrylate ends)	Visible blue-green light (450–500 nm); eosin Y + triethanolamine; in situ photopolymerization	Sealant without a drug (adjunct to tissue closure)	FDA-approved (2000) for air leaks after lung resection; clinically, leak-free fraction at discharge 39% vs. 11%; hydrolysis ~8 weeks	[88]
GelrinC (PEG diacrylate + denatured fibrinogen)	UV, in situ photopolymerization (PEG-fibrinogen platform, usually Irgacure 2959)	Acellular scaffold (drug-free)	FDA IDE pivotal study vs. microfracture (NCT03262909); MRI MOCART/T2–improved regenerate up to 24 months; resorption 6–12 months	[90,91]
Corneal crosslinking (native collagen crosslinking)	UV-A; riboflavin as a photoenhancer	Riboflavin (crosslinking agent, not a depot)	FDA-approved (Photrexa/KXL, 2016); clinical standard for keratoconus	[86]
GelMA scaffolds and bioprinting	UV/visible light; LAP or Irgacure 2959; radical photopolymerization	Cells, growth factors (varies)	Preclinical basis for cartilage, bone, vascular, and skin engineering; no marketed product	[12]

**Table 7:** Photo-responsive in situ hydrogels in oncology.

System	Light and Mechanism	Drug/Agent	Model and Outcome	Ref.
Azide-lactose chitosan (Az-CH-LA)	UV/photocrosslinking	Paclitaxel	Lewis lung tumor (3LL), mice; inhibition of growth and angiogenesis	[93]
Glycol chitosan	Visible light/photocrosslinking	Paclitaxel/β-cyclodextrin	Ovarian cancer, mice; release over 7 days	[94]
PEG-PCL-Acr + PEG/MPEG	Visible light, 405 nm/photopolymerization (LAP)	Doxorubicin	In situ gelation in mice; in vitro activity	[10]
Fibrin gel	NIR 808 nm (NO release)	Doxorubicin + NO	Resection cavity; suppression of postoperative recurrence in mice	[95]
Gel with gold nanorods	NIR/photothermal	Doxorubicin	Release over more than a month; chemo-photothermal therapy	[54]
Hyaluronic acid + Au nanorods	NIR/photothermal (LSPR)	–(photothermal platform)	Local heating and release	[96]
BP@PLEL (black phosphorus)	NIR/photothermal	–	Sprayed onto the wound; ablation of residual tumor + antibacterial effect	[55]
DOX@polydopamine@P(NIPAAm-co-AM) in PLGA-PEG-PLGA	NIR/photothermal + thermogelation	Doxorubicin	Release up to 87%; tumor suppression 98.77%	[97]
Alginate + C_3_N_4_/Au	Visible light/photocatalysis	CO + FA-doxorubicin micelles	Breast cancer; CO_2_ → CO, enhancement of chemotherapy	[98]

**Table 8:** Critical quality attributes (CQAs) of photo-responsive in situ hydrogels, recommended characterization methods, and the associated critical process parameters.

Quality Attribute (CQA)	What It Reflects and Why It Matters	Characterization Method	Associated CPP/Risk	Ref.
Gelation time and point	Whether the system sets at the injection site	Real-time oscillatory photorheology (G′/G″ crossover)	Intensity, dose, initiator concentration	[18,58]
Degree of double-bond conversion	Completeness of crosslinking; residual unreacted groups	FTIR/Raman spectroscopy (C=C band), photo-DSC, NMR	Dose, initiator, oxygen	[58]
Cure depth and uniformity	Whether the entire depot is crosslinked; absence of an undercured core	Cure-depth measurement, FTIR mapping, optical inspection	Dose, initiator absorption, wavelength, oxygen	[67,68]
Elastic modulus and viscoelasticity	Mechanical match to tissue; network density	Oscillatory rheology (G′), compression testing, AFM nanoindentation	Initiator and polymer concentration, dose	[18,19]
Porosity and mesh size	Diffusion and release rate; cell infiltration	SEM/cryo-SEM, swelling-based estimation, micro-CT	Crosslinking density	[10,78]
Swelling ratio	Dimensional stability; indirectly, network density	Gravimetric swelling test	Crosslinking density	[10,58]
Degradation rate	Depot lifetime; synchrony with release	Mass loss (gravimetry), enzymatic degradation, in vivo	Crosslinking density, polymer chemistry	[10,81]
Loading and encapsulation efficiency	Actual drug dose in the depot	Extraction followed by HPLC/UV analysis	Material characteristics, drug compatibility	[10]
Release profile (including burst)	Duration of therapy and dosing safety	In vitro release test (compendial apparatus/dialysis) + HPLC/UV	Mesh size, crosslinking density	[81,97]
Injectability (rheology before crosslinking)	Ability to deliver through a fine needle	Injection-force test, viscosity measurement	Polymer concentration and molecular weight	[10,95]
Residual monomer/macromer and initiator	Direct source of toxicity	HPLC, LC-MS	Dose, degree of conversion	[66]
Oxidative load (ROS)	Mechanism of photosystem cytotoxicity	Fluorescent ROS assays (e.g., DCFH-DA)	Type and concentration of initiator, dose	[19]
Cytocompatibility	Biological safety	ISO 10993-5 (viability tests, live/dead staining)	Initiator, dose, residuals, ROS	[19,101]
Sterility and endotoxins	Safety of injectable administration	Sterility test, LAL endotoxin test	Manufacturing and sterilization	[100]
Stress relaxation/viscoelasticity	Time-dependent mechanics, regulating cell behavior and drug release	Stress-relaxation test, oscillatory rheology (tan δ)	Crosslinking density, dynamic bonds	[63,64]

## Data Availability

No new data were created or analyzed in this study. Data sharing is not applicable to this article.

## Abbreviations

The following abbreviations are used in this manuscript:

3D	Three-dimensional
4D	Four-dimensional
3LL	Lewis lung carcinoma tumor model
ADA	American Dental Association
AFM	Atomic force microscopy
Az-CH-LA	Azide- and lactose-modified chitosan
BP	Black phosphorus
BP@PLEL	Black-phosphorus-loaded PLEL hydrogel
CMA	Critical material attribute
CQA	Critical quality attribute
CPP	Critical process parameter
CXL	Corneal crosslinking
DA	Diels–Alder
DA-PF127	Diacrylated Pluronic F127
DCFH-DA	2′,7′-Dichlorodihydrofluorescein diacetate
DLP	Digital light processing
DOI	Digital object identifier
DOX	Doxorubicin
DPN	Doxorubicin-loaded polydopamine/poly(N-isopropylacrylamide-co-acrylamide) nanoparticles
DS	Degree of substitution
DSC	Differential scanning calorimetry
ECM	Extracellular matrix
EMA	European Medicines Agency
FA	Folic acid
FDA	United States Food and Drug Administration
FD&C	Federal Food, Drug, and Cosmetic
FibMA	Methacrylated fibrinogen
FTIR	Fourier-transform infrared spectroscopy
G4-PAMAM	Generation-4 poly(amidoamine) dendrimer
GelMA	Gelatin methacryloyl
GelMA-Z	ZIF-8/GelMA composite hydrogel
GelNB	Gelatin norbornene
Gel-Phe	Phenyl-isothiocyanate-modified gelatin
GG	Gellan gum
GGMA	Methacrylated gellan gum
HA	Hyaluronic acid
HAMA	Hyaluronic acid methacrylate
HPLC	High-performance liquid chromatography
I-2959	Irgacure 2959
ICH	International Council for Harmonisation of Technical Requirements for Pharmaceuticals for Human Use
IDE	Investigational Device Exemption
IPN	Interpenetrating polymer network
ISO	International Organization for Standardization
LAL	Limulus amebocyte lysate
LAP	Lithium phenyl-2,4,6-trimethylbenzoylphosphinate
LC–MS	Liquid chromatography–mass spectrometry
LED	Light-emitting diode
LSPR	Localized surface plasmon resonance
MDR	Medical Device Regulation
MeGG	Methacrylated gellan gum
MeHA	Methacrylated hyaluronic acid
micro-CT	Micro-computed tomography
MMP	Matrix metalloproteinase
MOCART	Magnetic Resonance Observation of Cartilage Repair Tissue
MPEG	Monomethoxy poly(ethylene glycol)
MRI	Magnetic resonance imaging
NIR	Near-infrared
NMR	Nuclear magnetic resonance
NVP	N-Vinylpyrrolidone
OMA	Oxidized methacrylated alginate
PAA	Poly(acrylic acid)
PACK-CXL	Photoactivated chromophore for infectious keratitis–corneal crosslinking
PAM	Polyacrylamide
PAMAM	Poly(amidoamine)
PCL	Poly(ε-caprolactone)
PDLLA	Poly(D,L-lactide)
PEG	Poly(ethylene glycol)
PEGDA	Poly(ethylene glycol) diacrylate
PEG-PCL-Acr	Acrylate-terminated PEG-PCL copolymer
PEO	Poly(ethylene oxide)
PGA	Poly(glycolic acid)
PLA	Polylactide
PLEL	Poly(D,L-lactide)-poly(ethylene glycol)-poly(D,L-lactide)
PLGA	Poly(lactic-co-glycolic acid)
PMCID	PubMed Central identifier
PMID	PubMed identifier
P(NIPAAm-co-AM)	Poly(N-isopropylacrylamide-co-acrylamide)
PPO	Poly(propylene oxide)
PRIME	Priority Medicines scheme
PVA	Poly(vinyl alcohol)
QbD	Quality by Design
QTPP	Quality target product profile
RGD	Arginine–glycine–aspartic acid
RGDS	Arginine–glycine–aspartic acid–serine
RGX	Rose bengal/green-light crosslinking
RMAT	Regenerative Medicine Advanced Therapy
ROS	Reactive oxygen species
Ru(bpy)_3_^2+^	Tris(2,2′-bipyridine)ruthenium(II)
SEM	Scanning electron microscopy
SLA	Stereolithography
SPS	Sodium persulfate
TEA	Triethanolamine
UCNP	Upconversion nanoparticle
UV	Ultraviolet
UV-A	Ultraviolet A
VC	N-Vinylcaprolactam
ZIF	Zeolitic imidazolate framework
ZIF-8	Zeolitic imidazolate framework-8
